# Soft Matter Electrolytes: Mechanism of Ionic Conduction Compared to Liquid or Solid Electrolytes

**DOI:** 10.3390/ma17205134

**Published:** 2024-10-21

**Authors:** Kyuichi Yasui, Koichi Hamamoto

**Affiliations:** National Institute of Advanced Industrial Science and Technology (AIST), Nagoya 463-8560, Japan; k-hamamoto@aist.go.jp

**Keywords:** soft matter electrolytes, polymer electrolytes, polymeric or inorganic gel electrolytes, VFT (Vogel-Fulcher-Tammann)-type behavior, free volume model, configurational entropy model, cavitation under tensile deformation, microporous structure, crystalline vs. amorphous phases, merits and demerits

## Abstract

Soft matter electrolytes could solve the safety problem of widely used liquid electrolytes in Li-ion batteries which are burnable upon heating. Simultaneously, they could solve the problem of poor contact between electrodes and solid electrolytes. However, the ionic conductivity of soft matter electrolytes is relatively low when mechanical properties are relatively good. In the present review, mechanisms of ionic conduction in soft matter electrolytes are discussed in order to achieve higher ionic conductivity with sufficient mechanical properties where soft matter electrolytes are defined as polymer electrolytes and polymeric or inorganic gel electrolytes. They could also be defined by Young’s modulus from about $10^{5}$ Pa to $10^{9}$ Pa. Many soft matter electrolytes exhibit VFT (Vogel–Fulcher–Tammann) type temperature dependence of ionic conductivity. VFT behavior is explained by the free volume model or the configurational entropy model, which is discussed in detail. Mostly, the amorphous phase of polymer is a better ionic conductor compared to the crystalline phase. There are, however, some experimental and theoretical reports that the crystalline phase is a better ionic conductor. Some methods to increase the ionic conductivity of polymer electrolytes are discussed, such as cavitation under tensile deformation and the microporous structure of polymer electrolytes, which could be explained by the conduction mechanism of soft matter electrolytes.

## 1. Introduction

In Li-ion batteries, liquid electrolytes, which are organic solutions of Li salts, have been widely used because of their high ionic conductivity and their excellent contact at the electrodes’ surfaces [1,2,3]. However, there is the safety problem that the organic solutions could possibly leak and become burnable upon heating by vaporization of the organic solvents [2,4]. In order to solve this safety problem, solid electrolytes have been studied because they never leak and are not burnable [4,5]. However, solid electrolytes have the problem of poor contact at the electrodes’ surfaces [5,6]. Soft matter electrolytes could simultaneously solve the problems of safety and poor contact [7,8]. When soft matter electrolytes are sufficiently rigid, separators used in Li-ion batteries are not necessary to prevent direct contact between anode and cathode materials [9]. However, their ionic conductivity is relatively low when their mechanical properties are relatively good for the application in Li-ion batteries [9]. In the present review, mechanisms of ionic conduction in soft matter electrolytes are discussed in order to achieve higher ionic conductivity with sufficient mechanical properties. Although there is a recent excellent review on polymer electrolytes [9], the mechanisms of ionic conduction are not the focus.

**Figure 1:**
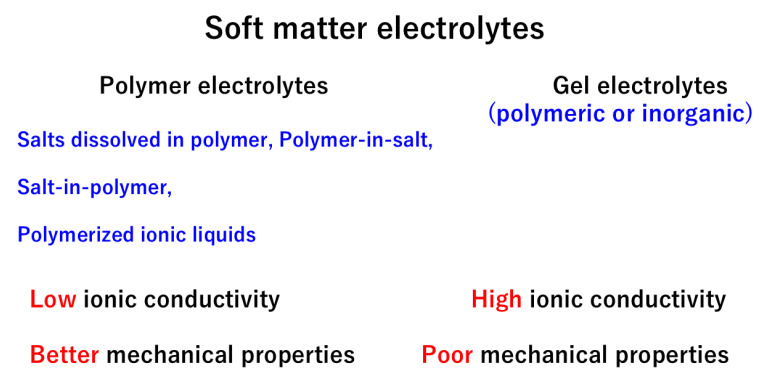
Soft matter electrolytes defined in the present review.

In the present review, soft matter electrolytes are defined as polymer electrolytes and polymeric or inorganic gel electrolytes in which there is considerable conduction of ${\mathrm{Li}}^{+}$ cations under an applied electric field for possible application to Li-ion batteries (Figure 1). Polymer electrolytes are mixtures of a polymer and Li salt(s) or polymerized ionic liquids [10,11,12,13,14,15,16,17,18,19,20,21,22]. Polymerized ionic liquids are produced by polymerization of organic ionic liquids [15,16]. Gel electrolytes are mostly swollen polymer electrolytes with organic or aqueous solvents as well as ionic liquid [23,24,25,26,27,28,29,30,31]. In some cases, gel electrolytes consist of inorganic networks swollen with aqueous or organic Li salt solution [32,33,34]. The softness of the materials may be expressed by Young’s modulus, with a lower value corresponding to a softer material [35]. Young’s modulus is defined as the ratio of stress to strain [36,37]. For polymer/gel electrolytes, it ranges from about $10^{5}$ Pa to $10^{9}$ Pa [6,38,39,40,41], which is lower than that of solid electrolytes (ceramics), ranging from about $10^{10}$ Pa to $10^{11}$ Pa [42]. The bulk modulus of liquid electrolytes is about $10^{9}$ Pa, which is estimated from the sound speed and density of the liquid [43,44].

**Figure 2:**
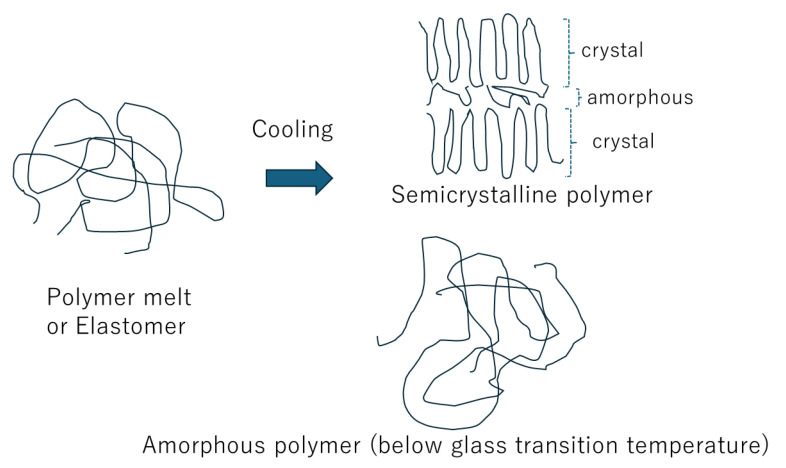
Change in the structure of a polymer with decreasing temperature.

As there is some distribution in the length of the polymer chains, amorphous regions appear between the crystal regions of a polymer, as shown in the upper right side of Figure 2, which is called a semicrystalline polymer [45,46,47]. In other words, polymer melt becomes a semicrystalline polymer upon cooling for some polymers. However, under some cooling conditions, they become an amorphous polymer (lower right side of Figure 2). For some other polymers, elastomer becomes an amorphous polymer upon cooling below glass transition temperature ($T_{g}$) [47]. It has long been believed that ionic conduction mostly occurs in the amorphous phase of a polymer, usually at a higher temperature than $T_{g}$ [10,11,12,13,14,48,49]. However, recently, it has been shown both experimentally and theoretically that ionic conductivity in crystalline polymer could be greater than that in equivalent amorphous material above $T_{g}$ for some cases [50,51,52]. This is discussed in the present review.

**Figure 3:**
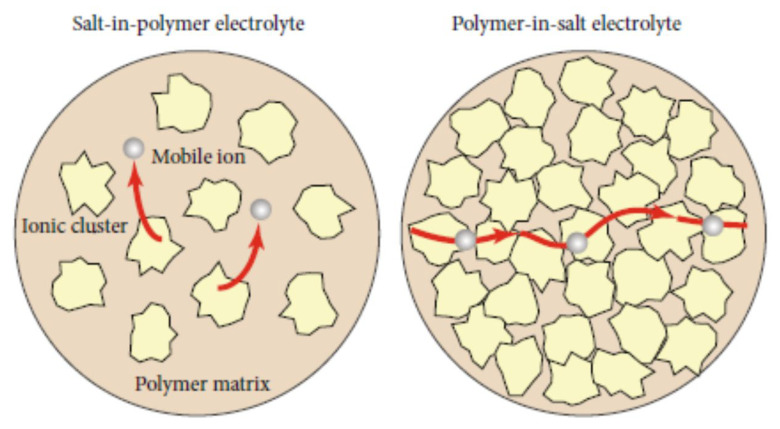
Schematic illustration of lithium-ion transport in a salt-in-polymer electrolyte and a polymer-in-salt electrolyte. Reprinted with permission from Ref. [56]. Copyright 2021, Hongcai Gao et al.

**Figure 4:**
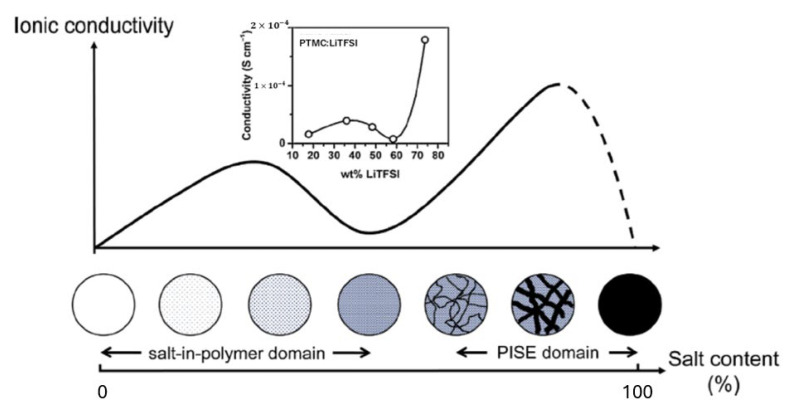
Schematic illustration of ionic conductivity as a function of salt concentration with the suggested morphology of salt-in-polymer electrolytes and polymer-in-salt electrolytes (PISE). The inset shows the data for the PTMC:LiTFSI system where PTMC is poly(trimethylene carbonate): ${\left(C_{4}H_{6}O_{3}\right)}_{n}$ and LiTFSI is lithium bis(trifluoromethanesulfonyl)imide: ${\mathrm{LiC}}_{2}F_{6}{\mathrm{NO}}_{4}S_{2}$. Reprinted with permission from Ref. [57]. Copyright 2018, Elsevier.

For some mixtures of a polymer and a Li salt, a Li salt dissolves in a polymer matrix at a molecular level like a Li salt dissolved in an organic solvent [53,54,55]. Other mixtures of a polymer and a Li salt(s) are categorized into salt-in-polymer electrolytes and polymer-in-salt electrolytes as shown in Figure 3 [56]. A schematic illustration of ionic conductivity as a function of salt concentration is shown in Figure 4 for such solid polymer electrolytes [57]. Highly conductive compositions can often be found where these materials are often amorphous, and the polymer itself merely plasticizes the salt crystals to the extent that the ions display a liquid-like behavior [57]. Polymer-in-salt electrolytes (PISE) often display higher conductivities than conventional solid polymer electrolytes, but their mechanical properties are too poor to be applied in devices [57].

Typical polymer electrolytes consist of poly(ethylene oxide) (PEO) and Li salts [10,11,12,13,14,58,59,60,61]. PEO typically has a molecular weight larger than about $2\times 10^{4}$ with the structure of $HO-{\left[CH_{2}-CH_{2}-O\right]}_{n}-H$ where n>450. PEO-based polymer electrolytes have an ionic conductivity of about $10^{-5}-10^{-3}$ S ${\mathrm{cm}}^{-1}$ at moderate temperatures [60]. One of the problems of PEO-based polymer electrolytes is their crystallization because high ionic conductivity is attributed primarily to ionic conduction in amorphous regions [60,62]. Some PEO-based polymer electrolytes form semicrystalline complexes with salts, which have a considerably higher melting point (~180 °C) than pure semicrystalline PEO (~60 °C) [62]. In order to make amorphous polymer electrolytes, poly(propylene oxide) (PPO)-based polymers are used [62]. The ionic conductivity of PPO-based polymer electrolytes is lower than or comparable with that of the PEO-based ones [62]. PPO has the structure of $HO-{\left[CH\left(CH_{3}\right)-CH_{2}-O\right]}_{n}-H$.

**Figure 5:**
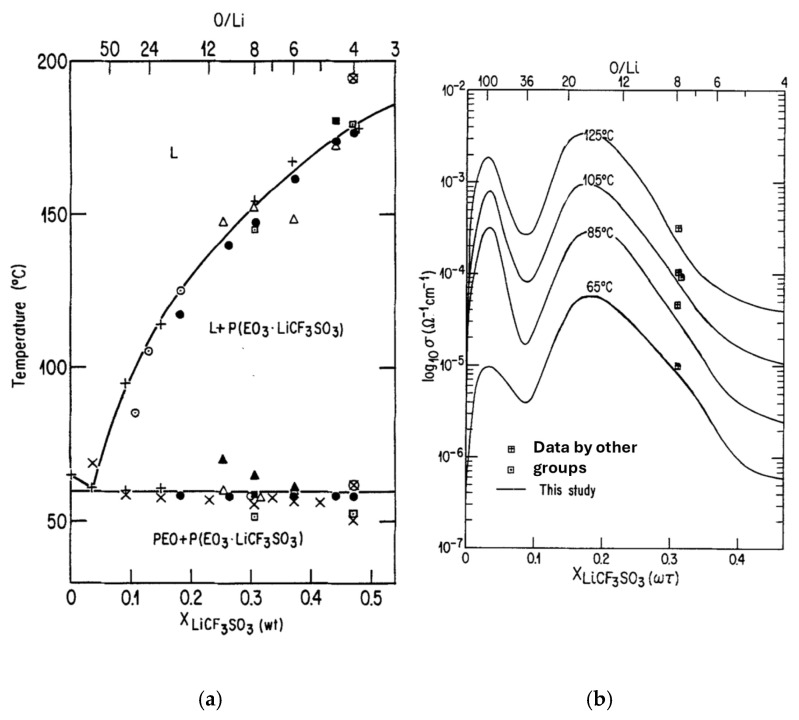
(**a**) Phase diagram of the PEO-${\mathrm{LiCF}}_{3}{\mathrm{SO}}_{3}$ system. The transition temperatures were obtained using various experimental techniques; NMR ⊡, DTA or DSC ● △ ⊗, conductivity ○ ▲ ×, optical microscopy ■ +, and modeling ⦿. (**b**) Isotherms of ionic conductivity (σ) in logarithmic scale vs. mass fraction (X) in weight of ${\mathrm{LiCF}}_{3}{\mathrm{SO}}_{3}$ in the electrolyte. Reprinted with permission from Ref. [63]. Copyright 1986, IOP Publishing Ltd.

As an example, the phase diagram of a PEO-based polymer electrolyte with ${\mathrm{LiCF}}_{3}{\mathrm{SO}}_{3}$ as a salt is shown in Figure 5a [63]. In the phase diagram, the molecular weight of PEO is about $9\times 10^{5}$, which corresponds to $n\approx 2\times 10^{4}$. When the mole fraction of O/Li is about 40, the sample contains a large proportion of eutectic mixture of PEO and $P\left({\mathrm{EO}}_{3}\cdot {\mathrm{LiCF}}_{3}{\mathrm{SO}}_{3}\right)$ with spherulitic morphology, and there is no melting below 55 °C. By 59 °C, two-thirds of the sample PEO_36_LiCF_3_SO_3_, which is a mixture of PEO and $P\left({\mathrm{EO}}_{3}\cdot {\mathrm{LiCF}}_{3}{\mathrm{SO}}_{3}\right),$ has melted. Around 60 °C, almost all the sample has melted, leaving a liquid of the eutectic composition in equilibrium with a small amount of the intermediate compound, whose spherulitic macrostructure is still conserved. The eutectic composition of PEO and $P\left({\mathrm{EO}}_{3}\cdot {\mathrm{LiCF}}_{3}{\mathrm{SO}}_{3}\right)$ was observed at around an O/Li mole ratio of 100, as seen in Figure 5a [63]. At the eutectic composition, the polymer electrolyte immediately melts above the melting point without leaving the intermediate compound.

The corresponding isotherms of ionic conductivity in logarithmic scale vs. mass fraction of ${\mathrm{LiCF}}_{3}{\mathrm{SO}}_{3}$ in the polymer electrolyte are shown in Figure 5b for T>60 °C [63]. Above 60 °C, the PEO-$P\left({\mathrm{EO}}_{3}\cdot {\mathrm{LiCF}}_{3}{\mathrm{SO}}_{3}\right)$ system has a two-phase composition: semicrystalline and amorphous, with ion mobility occurring mainly in the amorphous regions. In Figure 5b, two conductivity maxima are observed: one at X≈0.03 (O/Li ≈100), the other at X≈0.16 (O/Li ≈18). The former coincides with the eutectic composition. Although the latter may coincide with the maximum proportion of amorphous regions, the mechanism for the latter maximum is unclear.

**Figure 6:**
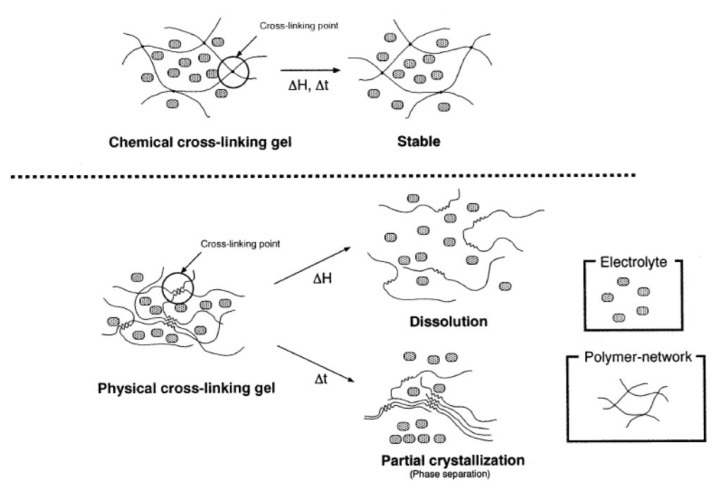
Models of gel electrolytes. Reprinted with permission from Ref. [24]. Copyright 2000, Elsevier.

Next, gel electrolytes are briefly discussed. A gel is defined as a system consisting of a polymer or inorganic network swollen with a solvent [45,47]. Most of the gel electrolytes studied until now are polymer-based gels [23,24,25,26,27,28,29,30,31]. As inorganic material-based gel electrolytes, lithium aluminosilicate gel (${\mathrm{Al}}_{2}O_{3}-{\mathrm{SiO}}_{2}-{\mathrm{Li}}_{2}O$ gel swollen with water) [33] and ${\mathrm{SiO}}_{2}$-based gel [32] are known. For polymer-based gel electrolytes, there are two categories: one with chemical cross-linking, and the other with physical cross-linking (Figure 6) [24]. The PEO-based gel is an example of the former, and PMMA (poly(methyl methacrylate): ${\left(C_{5}O_{2}H_{8}\right)}_{n}$)-based gel is an example of the latter. The chemical cross-linking gels are mostly thermally stable, but the physical cross-linking ones could dissolve under heating [24]. Furthermore, the solution could be gradually leaked from the physical cross-linking ones (Figure 6) [24]. For some gel electrolytes, relatively high ionic conductivity has been achieved [25,64]. However, the mechanical properties of gel electrolytes are rather poor.

In the present review, the mechanism of ionic conduction in soft matter electrolytes is discussed through the temperature dependence of ionic conductivity in order to achieve higher ionic conductivity with sufficient mechanical properties. Then, the problem of whether the amorphous or crystalline phase of polymer electrolytes is a better ionic conductor is discussed. Several possible methods to increase the ionic conductivity of polymer electrolytes are discussed, such as cavitation under tensile deformation and microporous structures, as well as the introduction of dislocations into the crystalline phase in an analogy with all-dislocation-ceramics proposed by the authors for ceramic solid electrolytes. Finally, the merits and demerits of soft matter electrolytes are summarized, comparing them with liquid and solid electrolytes.

## 2. Temperature Dependence of Ionic Conductivity

**Figure 7:**
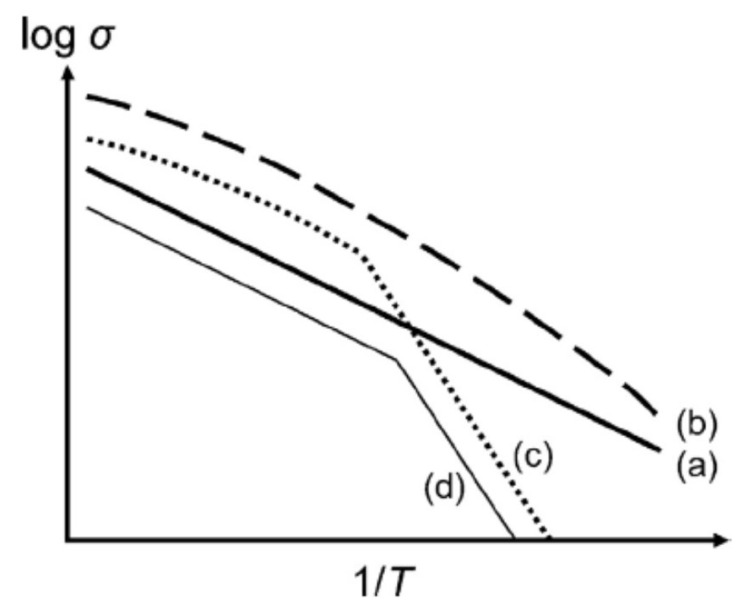
Schematic illustration of ionic conductivity as a function of reciprocal temperature. (a) Arrhenius behavior; (b) VFT behavior; (c) typical behavior of semi-crystalline polymers (such as PEO-based systems), where melting of the crystalline phase occurs after which VFT behavior is displayed; (d) behavior of crystalline systems where a solid–solid phase transition occurs, e.g., ${\left(\mathrm{PEO}\right)}_{8}{\mathrm{NaAsF}}_{6}$. Reprinted with permission from Ref. [57]. Copyright 2018, Elsevier.

There are two main types of temperature dependence of ionic conductivity for soft matter electrolytes as well as liquid or solid electrolytes: the Arrhenius type, and the VFT (Vogel–Fulcher–Tammann) type (Figure 7a,b) [14,57]. The Arrhenius type is described by Equation (1) or (2) [11,14,32,65,66,67,68,69,70,71,72,73].

(1) $$\sigma =\sigma_{0}e^{-E_{a}/k_{B}T}$$

(2) $$\sigma =\frac{{\sigma^{\prime }}_{0}}{T}e^{-{E^{\prime }}_{a}/k_{B}T}$$

where σ is ionic conductivity, $\sigma_{0}$ and ${\sigma^{\prime }}_{0}$ are constants, $E_{a}$ and ${E^{\prime }}_{a}$ are activation energies, T is absolute temperature, and $k_{B}$ is the Boltzmann constant. Equation (2) is derived by assuming the Arrhenius relation for the diffusion coefficient of ions (Equation (3)) [71,74].

(3) $$D=D_{0}e^{-{E^{\prime }}_{a}/k_{B}T}$$

where D is the diffusion coefficient of ions and $D_{0}$ is a constant. Using the following Nernst–Einstein equation [74], Equation (2) is obtained.

(4) $$D=\frac{\sigma k_{B}T}{Nq^{2}}$$

where N is the particle density of the charge carriers and q is their charge. Accordingly, the following relationship is obtained.

(5) $${\sigma^{\prime }}_{0}=\frac{Nq^{2}D_{0}}{k_{B}}$$

In Equation (2), however, the temperature dependence is mainly by the exponential factor, which is nearly the same as that in Equation (1).

The VFT type is described by Equation (6) or (7) [10,14,57,68,75,76,77,78,79,80,81,82,83].

(6) $$\sigma =AT^{-\frac{1}{2}}e^{-B/k_{B}\left(T-T_{0}\right)}$$

(7) $$\sigma =A^{\prime }e^{-B^{\prime }/k_{B}\left(T-T_{0}\right)}$$

where A and $A^{\prime }$ are constants, B and $B^{\prime }$ are constants with the dimension of energy, and $T_{0}$ is a reference temperature which is sometimes equivalent to the glass transition temperature ($T_{g}$). As in the case of the Arrhenius type, the temperature dependence of Equations (6) and (7) is mostly due to the exponential factor. Thus, Equations (6) and (7) are relatively similar.

There are several variations in the temperature dependence of ionic conductivity [57]. The plot of 1/T vs. logσ is called the Arrhenius plot because the plot becomes a straight line when the Arrhenius relationship (Equation (1)) holds. For Figure 7c, Arrhenius-type behavior changes at the melting point above which VFT behavior is observed, as in the case of PEO-based systems [57]. For Figure 7d, the activation energy for the Arrhenius relationship, which is related to the slope of the curve, is changed at the temperature of a solid–solid phase transition [57]. In summary, Arrhenius-type behavior is widely observed for crystalline or amorphous solid electrolytes as well as polymer electrolytes below the glass transition temperature and inorganic gel electrolytes [32,34,65,70,73,84,85]. On the other hand, VFT behavior is widely observed for liquid or polymeric gel electrolytes as well as polymer electrolytes above the glass transition temperature [23,65,68,77].

**Figure 8:**
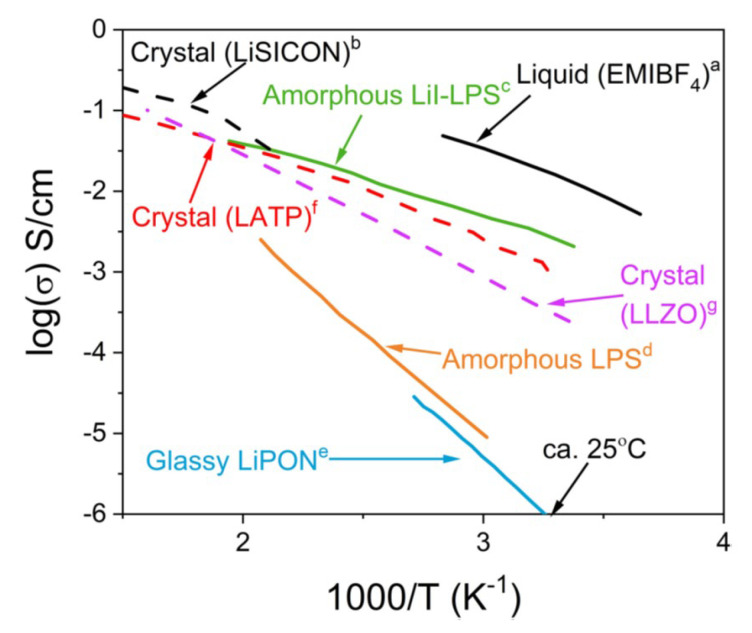
Ionic conductivity of a liquid electrolyte as well as crystalline or amorphous solid electrolytes as a function of reciprocal temperature. The data are from a [87], b [88], c [91], d [92], e [72], f [89], and g [90]. Reprinted with permission from Ref. [86]. Copyright 2020, Grady et al.

In Figure 8, the temperature dependence of ionic conductivity for some liquid electrolytes as well as crystalline or amorphous solid electrolytes is shown [86]. The liquid electrolyte shown in Figure 8 is 1-ethyl-3-methyl-imidazolium tetrafluoroborate (${\mathrm{EMIBF}}_{4}$), which is a room-temperature ionic liquid with a high ionic conductivity of about $10^{-2}{\;S\;\mathrm{cm}}^{-1}$ at room temperature, comparable to those of organic solvent electrolytes [87]. For ${\mathrm{Li}}^{+}$ ion conduction, Li salt is dissolved in the ionic liquid (${\mathrm{EMIBF}}_{4}$), which typically has a slightly lower ionic conductivity than that without salt (pure ${\mathrm{EMIBF}}_{4}$) [87]. As crystalline solid electrolytes, the experimental data for LiSICON, LATP, and LLZO are shown in Figure 8 [86]. LiSICON is named after Li superionic conductor and is ${\mathrm{Li}}_{14}\mathrm{Zn}{\left({\mathrm{GeO}}_{4}\right)}_{4}$ [88]. LATP and LLZO in Figure 8 are ${\mathrm{Li}}_{1.3}{\mathrm{Al}}_{0.3}{\mathrm{Ti}}_{1.7}{\left({\mathrm{PO}}_{4}\right)}_{3}$ and ${\mathrm{Li}}_{7}{\mathrm{La}}_{3}{\mathrm{Zr}}_{2}O_{12}$, respectively [89,90]. As amorphous solid electrolytes, the data for LiI-LPS, LPS, and LiPON are shown in Figure 8 [86]. LiI-LPS in Figure 8 is for amorphous ${\mathrm{LiPO}}_{3}-\mathrm{LiI}\;\left(\mathrm{mole}\;\mathrm{fraction}\;33\%\right)$ systems [91]. LPS in Figure 8 is for amorphous $70{\mathrm{Li}}_{2}S\cdot 30P_{2}S_{5}$ (mol%) [92]. LiPON is for amorphous ${\mathrm{Li}}_{x}{\mathrm{PO}}_{y}N_{z}$ [92]. In general, typical liquid electrolytes have higher ionic conductivities than those of solid or soft matter electrolytes [93]. For solid electrolytes, some amorphous ones have higher ionic conductivities than those of crystalline ones. However, for some other amorphous ones, ionic conductivities are lower than those of the crystalline ones, as seen in Figure 8 [86].

**Figure 9:**
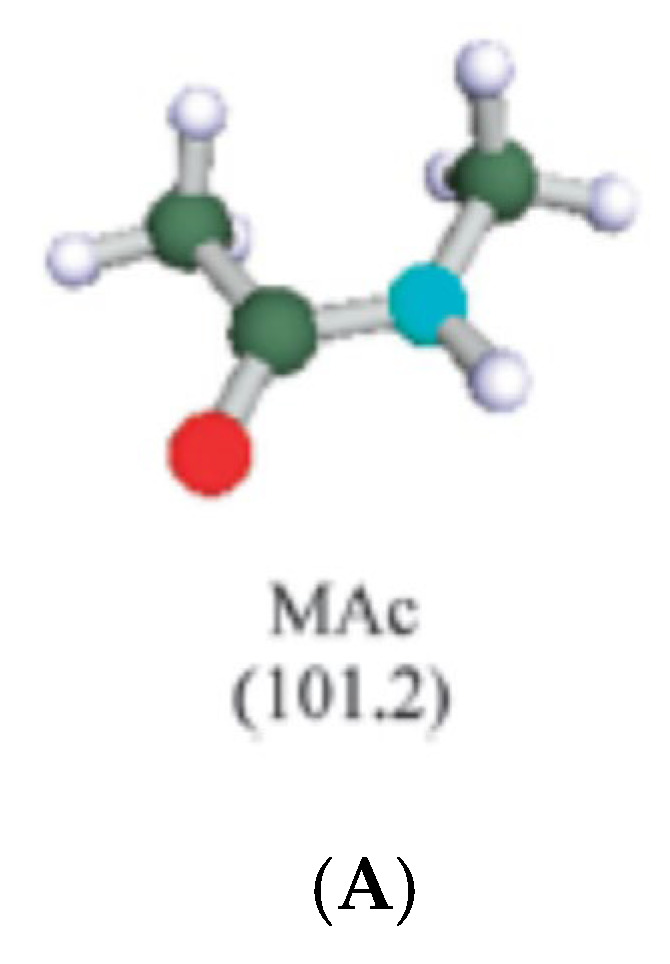

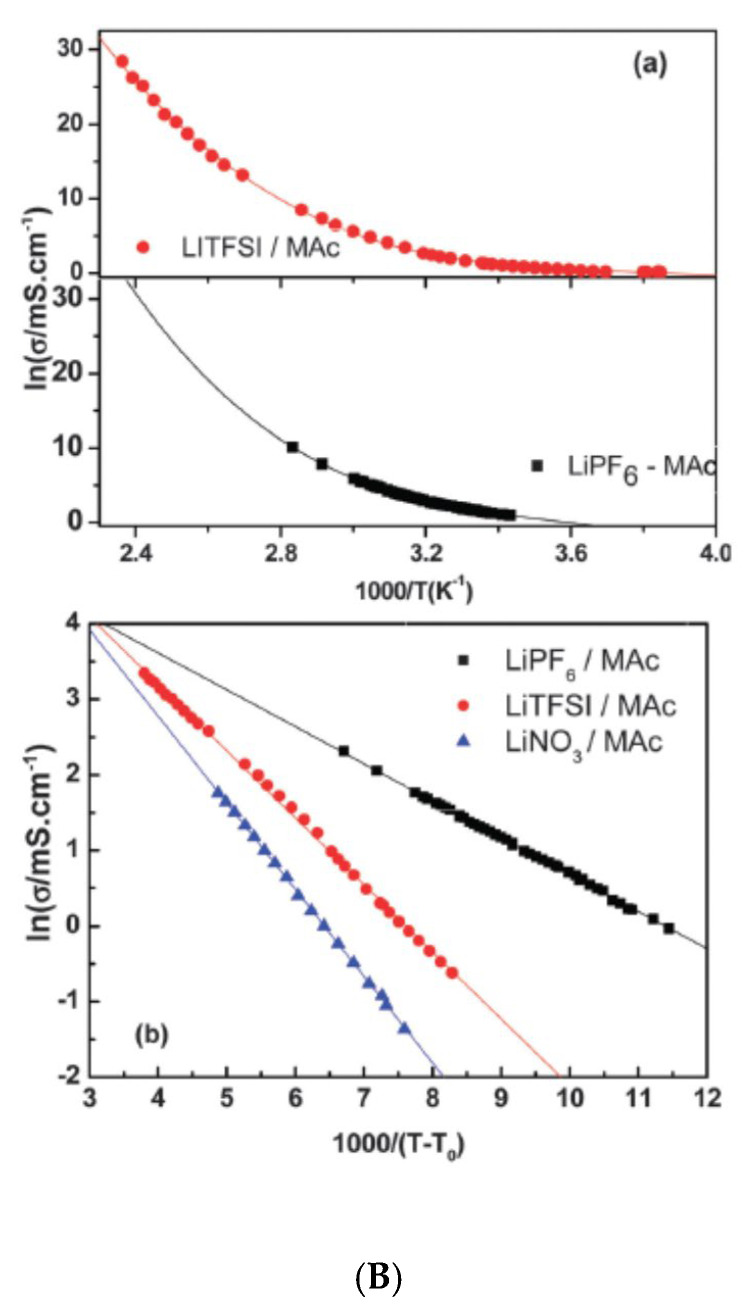
(**A**) Structure of N-methylacetamide (Mac) ($CH_{3}CONHCH_{3}$) with its volume in ${Å}^{3}$. C (green), H (white), N (blue), and O (red). (**B**) Arrhenius (**a**) and VFT (**b**) plots on the temperature dependence of ionic conductivity of liquid electrolytes (Mac with Li salts). The lithium-salt mole fraction was 0.2. The solid lines represent the VFT fitting. Reprinted with permission from Ref. [68]. Copyright 2013, Royal Society of Chemistry.

As already noted, VFT behavior is widely observed for liquid electrolytes, as seen in Figure 9, which shows the temperature dependence of ionic conductivities for N-methylacetamide (Mac) ($CH_{3}CONHCH_{3}$) with several Li salts [68]. In this case, the Arrhenius plots in Figure 9B(a) do not show straight lines, which implies that the Arrhenius relationship does not hold for them. On the other hand, the plots of 1/(T−T_0_) vs. lnσ(=$\log_{e}\sigma$) show straight lines, which implies that they are expressed by the VFT relationship (Equation (7)) [68].

**Figure 10:**
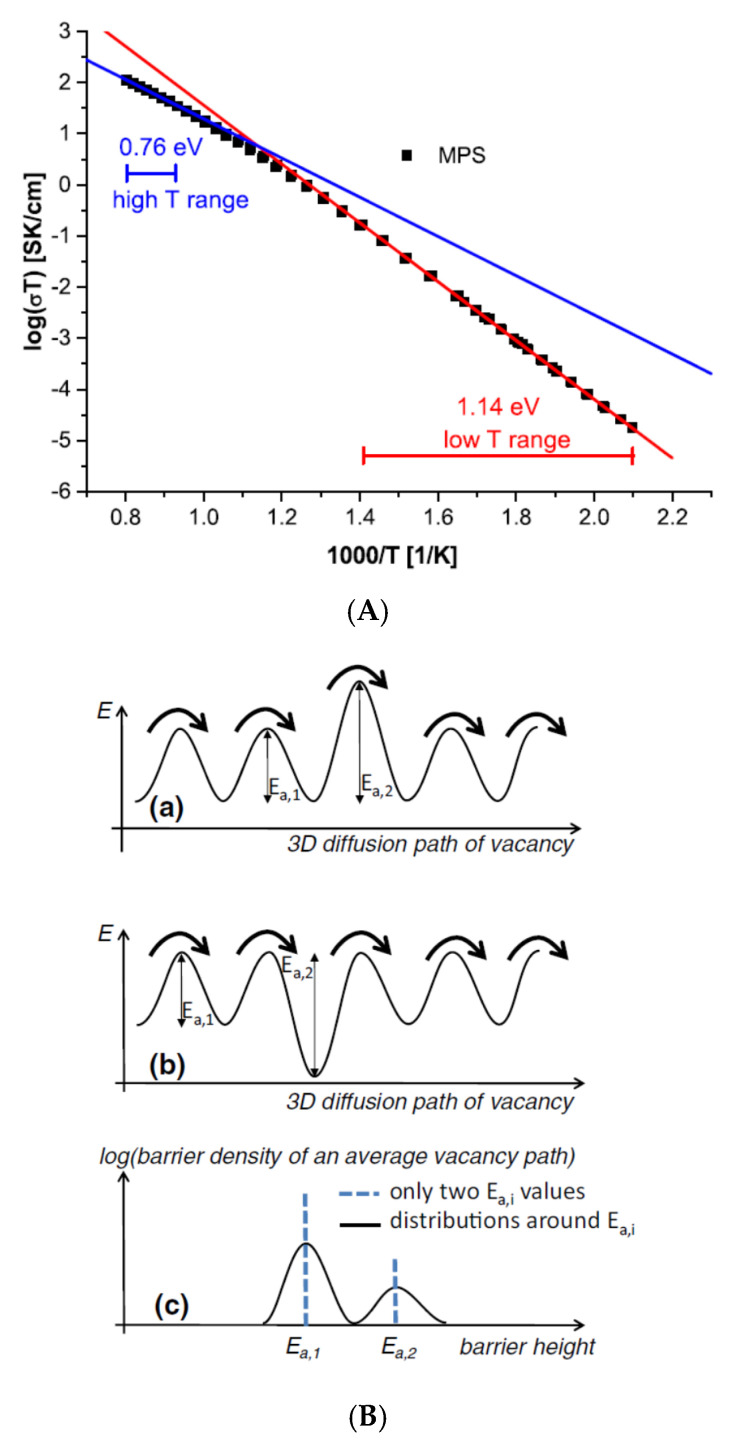
(**A**) Arrhenius plot of ionic conductivities measured for Yttria-stabilized Zirconia (YSZ) single crystal (solid electrolyte). MPS is a sample name. (**B**) (**a**) Sketch of a series of barriers with one energetically very unfavorable transition state. (**b**) Sketch of series of barriers with one energetically very favorable ground state. (**c**) Bimodal barrier distributions with exactly two barrier heights or a broad distribution of heights with two maxima. Reprinted with permission from Ref. [71]. Copyright 2017, Ahamer et al.

For some solid electrolytes (Yttria-stabilized Zirconia (YSZ) single crystal), the Arrhenius-like plot of 1/T vs. $\log\left(\sigma T\right)$ indicates that the activation energy at relatively high temperatures in Equation (2) is different from that at lower temperatures (Figure 10A) [71]. Ahamer et al. [71] suggested that the reason for this is the existence of two different activation energies in the 3D diffusion of oxygen vacancies by which ionic conduction occurs as follows (Figure 10B). The jump frequency ($\nu_{i}$) of a vacancy across the activation energy $E_{a,i}$ (the barrier height in Figure 10B) is given as follows:

(8) $$\nu_{i}=\nu_{i}^{0}e^{-E_{a,i}/k_{B}T}$$

where $\nu_{i}^{0}$ is a pre-factor. When there are two barrier heights ($E_{a,1}$ and $E_{a,2}$) as in Figure 10B, the effective jump frequency ($\nu_{eff}$) is given as follows [71]:

(9) $$\nu_{eff}=\frac{N_{jump}}{t}=\frac{\sum_{i}N_{jump,i}}{\sum_{i}N_{jump,i}\tau_{i}}=\frac{\sum_{i}N_{jump,i}}{\sum_{i}\frac{N_{jump,i}}{\nu_{i}}}$$

where $N_{jump}$ is the total number of successful jumps of a vacancy per time t, $N_{jump,i}$ is the number of successful jumps across barriers i (i= 1 or 2), and $\tau_{i}$ is the time needed until a successful jump takes place ($\tau_{i}=\nu_{i}^{-1}$). Then, the diffusion coefficient (D) of vacancies is expressed as follows [71]:

(10) $$D=a_{0}^{2}\nu_{eff}$$

where $a_{0}$ is the average jump distance which is half of the lattice constant of YSZ. From the Nernst–Einstein equation (Equation (4)), ionic conductivity is obtained as follows [71]:

(11) $$\sigma T={\left(\frac{1}{\gamma_{1}e^{-E_{a,1}/k_{B}T}}+\frac{1}{\gamma_{2}e^{-E_{a,2}/k_{B}T}}\right)}^{-1}$$

where Equations (8)–(10) have been used, and $\gamma_{1}$ and $\gamma_{2}$ are constants. If $\gamma_{2}\gg \gamma_{1}$ holds under $E_{a,1}<E_{a,2}$, temperature dependence of σT is mainly determined by $E_{a,1}$ at relatively high temperatures. For lower temperatures, it is determined mainly by $E_{a,2}$. The result agrees with the experimental data shown in Figure 10A [71]. When there is only one activation energy (barrier height), Equation (11) is reduced to the Arrhenius-type relationship (Equation (2)).

**Figure 11:**
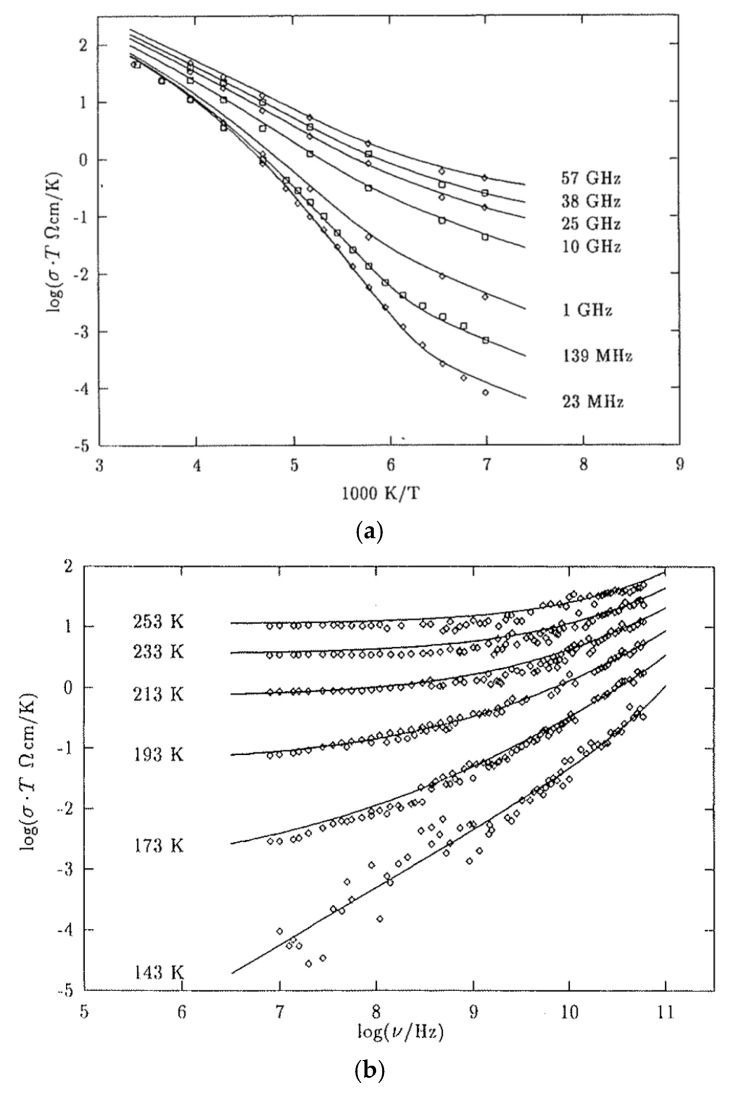
(**a**) Arrhenius-like plots of ionic conductivities of glass-forming molten salt $\mathrm{LiCl}\cdot 7H_{2}O$ above the glass transition temperature (139 K) for various frequencies of applied electric field. (**b**) The corresponding plots of ionic conductivities as a function of frequency for various constant temperatures. Reprinted with permission from Ref. [94]. Copyright 1995, Taylor & Francis Ltd.

It has been experimentally reported that temperature dependence of Ionic conductivity depends on the frequency of applied AC electric field (Figure 11) [94]. In Figure 11a, Arrhenius-like plots of ionic conductivities of glass-forming molten salt $\mathrm{LiCl}\cdot 7H_{2}O$ above the glass transition temperature are shown for various frequencies of applied AC electric field. Above the glass transition temperature, VFT behavior is observed at relatively low frequencies as expected (for the case of 23 MHz in Figure 11a) [94]. However, at much higher frequencies, the plots become nearly straight lines, which implies that the Arrhenius relation nearly holds. It suggests that ionic conduction at high frequencies is somewhat similar to that in solid or glass electrolytes even above the glass transition temperature [94]. The corresponding plots of ionic conductivities as a function of frequency are shown in Figure 11b for various constant temperatures [94].

**Figure 12:**
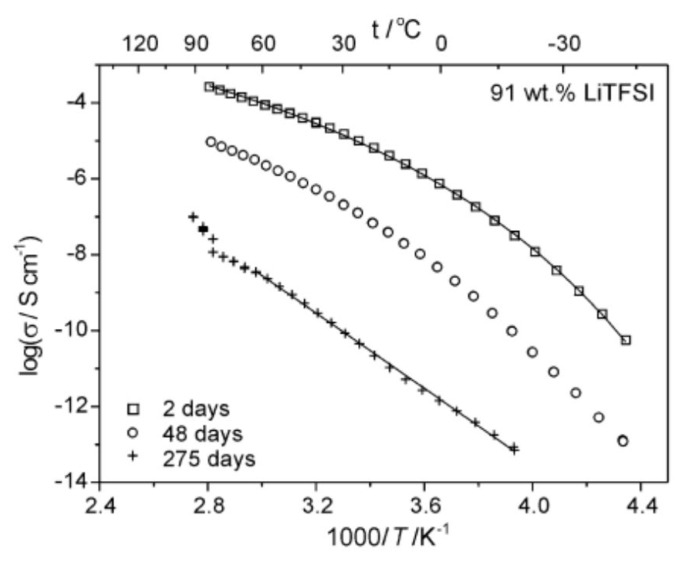
Effect of aging on Arrhenius plot of ionic conductivities of polymer electrolyte composed of an acrylonitrile and butyl acrylate copolymer with addition of 91 wt% of $\mathrm{LiN}{\left({\mathrm{CF}}_{3}{\mathrm{SO}}_{2}\right)}_{2}$ (LiTFSI). The solid lines represent the VFT fitting (for freshly cast film) and the Arrhenius fitting (for samples stored for 275 days). Reprinted with permission from Ref. [95]. Copyright 2015, Elsevier.

It has also been experimentally reported that the type of temperature dependence of ionic conductivity could change with aging (Figure 12) [95]. In Figure 12, Arrhenius plots of ionic conductivity of polymer-in-salt electrolytes are shown for various storage days of the sample at room temperature in argon atmosphere. For freshly cast film, VFT behavior was observed above the glass transition temperature as expected [95]. However, for the sample stored for 275 days, the behavior was changed to the Arrhenius type even above the glass transition temperature, which was considerably increased, as seen in Figure 12 [95]. The absolute value of ionic conductivity considerably decreased after the long storage time. This suggests that the polymer-in-salt electrolyte became a solid-like electrolyte even above the glass transition temperature after considerable aging [95]. One of the causes of the aging may be the precipitation of the salt in the polymer electrolyte [95]. Another possibility is the gradual change of coordination environment of lithium cations leading to the cross-linking of polymer chains, which suppresses ionic conductivity [95].

## 3. Mechanism for Ionic Conduction (Theory)

As discussed in the previous section, there are two main types of temperature dependence of ionic conductivities for soft matter electrolytes as well as solid or liquid ones: the Arrhenius type, and the VFT type. For the VFT type, there are two main theoretical models to explain the behavior: the free volume model, and the configurational entropy model [10]. In the present section, these models are discussed. For the Arrhenius type, it is explained by the jump frequency of diffusion for ions or vacancies, as already discussed in the previous section. In the present section, the jump-diffusion model is also briefly discussed.

### 3.1. Free Volume Model

Firstly, the viscosity of liquid or polymer above the glass transition temperature (elastomer or liquid) is discussed. It has been discussed that viscosity decreases as the average free volume per molecule increases [96]. The average free volume per molecule ($v_{f}$) is defined as follows [97].

(12) $$v_{f}=\bar{v}-v_{0}$$

where $\bar{v}$ is the average volume per molecule in the liquid or elastomer, and $v_{0}$ is the van der Waals volume of the molecule. In 1951, Doolittle [96] found that viscosity (η) is related to the average free volume per molecule ($v_{f}$) as follows.

(13) $$\eta =\alpha e^{bv_{0}/v_{f}}$$

where α and b are constants. Diffusion coefficient (D) of ions is related to viscosity (η) as follows, according to the Stokes–Einstein relation [97,98].

(14) $$D=\frac{k_{B}T}{6\pi a\eta }$$

where a is the radius of the molecule. Using the Nernst–Einstein equation (Equation (4)), the following relationship is obtained from Equations (13) and (14).

(15) $$\sigma =A^{\prime }e^{-bv_{0}/v_{f}}$$

where $A^{\prime }$ is a constant. In 1959, Cohn and Turnbull [97] suggested that the average free volume per molecule is related to temperature as follows.

(16) $$v_{f}=\alpha^{\prime }\left(T-T_{0}\right)$$

where $\alpha^{\prime }$ is a constant, and $T_{0}$ is a reference temperature which is sometimes equivalent to the glass transition temperature ($T_{g}$). From Equations (15) and (16), the VFT equation (Equation (7)) is derived.

**Figure 13:**
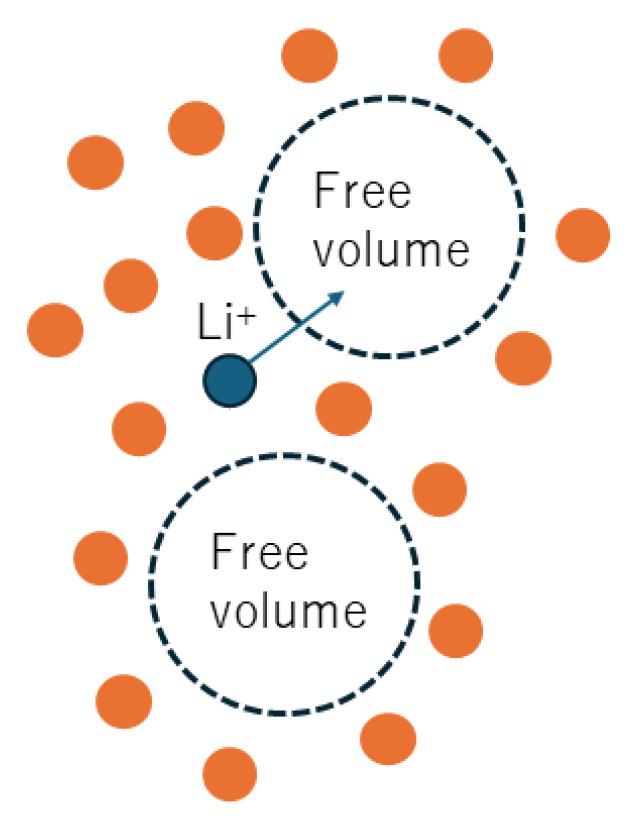
The free volume model.

This means that ionic conductivity increases as the average free volume per molecule increases with the increase in temperature above the glass transition temperature (Figure 13).

### 3.2. Configurational Entropy Model

In liquid or elastomer, ionic conduction occurs in association with some cooperative rearrangements of liquid or polymer molecules. The probability (W) for such cooperative rearrangements of molecules may be expressed as follows, as Adam and Gibbs [99] reported in 1965.

(17) $$W=A_{W}e^{-z^{*}\Delta \mu /k_{B}T}$$

where $A_{w}$ is a constant, $z^{*}$ is the minimum number of molecules (or monomeric segments in the case of polymers) involved in the cooperative rearrangements, and Δμ is the change of chemical potential of molecules in the cooperative rearrangements. Here, the molar configurational entropy ($S_{c}$) of the macroscopic supersystem is introduced as follows [99]:

(18) $$S_{c}=k_{B}\ln W_{c}$$

where $W_{c}$ is the number of configurations. Next, the configurational entropy ($s_{c}^{*}$) of a subsystem with the number of $z^{*}$ molecules is considered as follows [99]:

(19) $$s_{c}^{*}=\frac{z^{*}}{N_{A}}S_{c}=k_{B}\ln\left(W_{c}^{z^{*}/N_{A}}\right)$$

where $N_{A}$ is the Avogadro number, and Equation (18) has been used. Accordingly, $z^{*}$ is simply given as follows:

(20) $$z^{*}=N_{A}s_{c}^{*}/S_{c}$$

Then, Equation (17) becomes as follows [99]:

(21) $$W=A_{w}e^{-s_{c}^{*}\Delta \mu /k_{B}TS_{c}}$$

where Δμ is expressed in J per mole. The molar entropy of the macroscopic system ($S_{c}$) can be expressed as follows [76]:

(22) $$S_{c}=\int \frac{\Delta C_{p}}{T}dT$$

where the heat-capacity difference ($\Delta C_{p}$) is given as follows for glass forming polymers [76,100]:

(23) $$\Delta C_{p}=\frac{B_{c}}{T}$$

where $B_{c}$ is a constant. Then, Equation (21) becomes as follows [76]:

(24) $$W=A_{w}e^{-s_{c}^{*}\Delta \mu /k_{B}TB_{c}\left(\frac{1}{T_{0}}-\frac{1}{T}\right)}$$

If $s_{c}^{*}$ is independent of temperature, Equation (24) becomes as follows [76]:

(25) $$W=A_{w}e^{-B^{\prime }/k_{B}\left(T-T_{0}\right)}$$

where $B^{\prime }$ is a constant. If ionic conductivity (σ) is proportional to the probability (W) of the cooperative rearrangements of molecules, the VFT relationship (Equation (7)) is obtained [76].

The configurational entropy model is applicable for soft materials or liquids, as in the case of the free volume model. This suggests that the VFT relationship (Equation (6) or (7)) is mostly applicable to soft matter or liquid electrolytes.

### 3.3. Jump-Diffusion Model

As discussed in the previous section, when the barrier heights are nearly the same throughout the electrolyte in Figure 10B, the jump frequency of diffusion for a vacancy or an ion is expressed by Equation (8) with a single value of the barrier height ($E_{a}$). Then, the effective jump frequency in Equation (9) is equivalent to the jump frequency in Equation (8). Finally, the Arrhenius relation (Equation (2)) is obtained from Equation (10) and the Nernst–Einstein equation (Equation (4)). As already noted, the Arrhenius type is observed widely for crystalline or amorphous solid electrolytes, as well as polymer electrolytes below the glass transition temperature and inorganic gel electrolytes which are mechanically relatively hard [32,34,65,70,73,84,85].

## 4. Crystal vs. Amorphous

As briefly discussed in the Introduction, it has been widely believed that ionic conduction in solid polymer electrolytes occurs mostly in the amorphous (elastomer) phase, typically at higher temperatures than the glass transition temperature [10,11,12,13,14,48,49,101]. Berthier et al. [48] experimentally measured ionic conductivity and the relaxation curves of pulsed NMR signals as a function of temperature for $P\left({\mathrm{EO}}_{8}\cdot {\mathrm{LiCF}}_{3}{\mathrm{SO}}_{3}\right)$ and $P({\mathrm{EO}}_{10}\cdot \mathrm{NaI}$). From the relaxation curve of pulsed NMR signals [48], the relative amounts of the crystalline (hard) and elastomeric (soft) phases were obtained at each temperature. Below the melting point of P(EO), the coexistence of the following three phases was observed: a crystalline complex of P(EO) and salt, pure crystalline P(EO), and a smaller amount of the elastomeric (amorphous) phase. It was shown that ionic mobility is only present in the elastomeric (amorphous) phase [48]. Henderson and Passerini [101] experimentally showed that fully amorphous $P\left({\mathrm{EO}}_{6}\cdot {\mathrm{LiClO}}_{4}\right)$ polymer electrolyte has ionic conductivity about two orders of magnitude higher than that of the same isostructural crystalline polymer electrolyte. Xue et al. [49] reported the diffusion pathways and activation energies of ${\mathrm{Li}}^{+}$ ions in both crystalline and amorphous $P\left({\mathrm{EO}}_{3}\cdot {\mathrm{LiCF}}_{3}{\mathrm{SO}}_{3}\right)$ polymer electrolyte, as determined by the density functional theory (DFT) and ab initio molecular dynamics simulations. The determined activation energy in the amorphous phase is 0.6 eV, which is much lower than that (1.0 eV) for the low-barrier diffusion pathway in the crystalline phase [49]. This result supports the experimental results of higher ionic conductivity in the amorphous phase of $P\left({\mathrm{EO}}_{3}\cdot {\mathrm{LiCF}}_{3}{\mathrm{SO}}_{3}\right)$ than in the crystalline phase. Spěváćek et al. [102,103] reported that the crystalline and amorphous phases have the same local structure, based on the NMR spectra of $\left(\mathrm{PEO}\right):{\mathrm{LiCF}}_{3}{\mathrm{SO}}_{3}$ polymer electrolyte and DFT quantum-chemical calculations. Accordingly, they suggested that the important factors which make the ionic conductivity in the amorphous phase much higher than in the crystalline phase are either the higher mobility of the amorphous phase or the long-distance conformational arrangement of the crystalline phase [102,103]. However, the reason for the higher ionic conductivity in the amorphous phase is still unclear.

**Figure 14:**
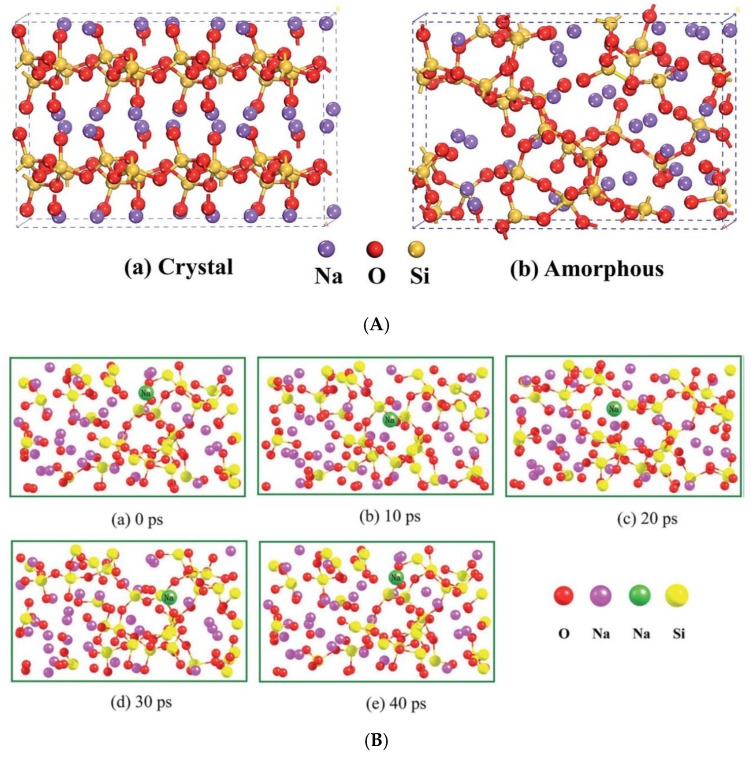

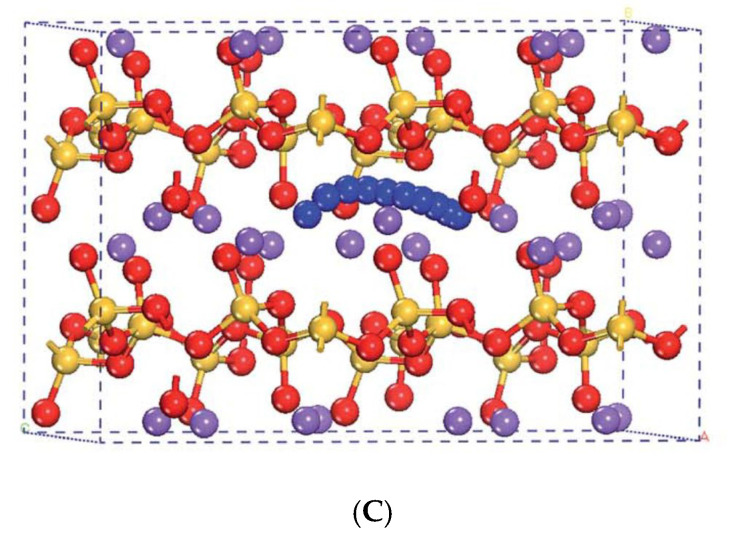
(**A**) The structures of crystalline (**a**) and amorphous (**b**) ${\mathrm{Na}}_{2}{\mathrm{Si}}_{2}O_{5}$ (solid electrolyte). (**B**) The ${\mathrm{Na}}^{+}$ transport in amorphous ${\mathrm{Na}}_{2}{\mathrm{Si}}_{2}O_{5}$ at 873 K for 40 ps by molecular dynamics simulation. The green ball represents ${\mathrm{Na}}^{+}$ in motion. The calculated energy barrier is 0.30 eV which enables fast ionic conduction. (**C**) The ${\mathrm{Na}}^{+}$ transport in crystalline ${\mathrm{Na}}_{2}{\mathrm{Si}}_{2}O_{5}$ by molecular dynamics simulation. The blue ball is the moving ${\mathrm{Na}}^{+}$. The calculated energy barrier is 1.18 eV, which is probably too high for a fast ${\mathrm{Na}}^{+}$ transport. Reprinted with permission from Ref. [104]. Copyright 2015, Royal Society of Chemistry.

Here, an ab initio molecular dynamics simulation by Lei et al. [104] is briefly reviewed to identify the reason for the much higher ionic conductivity in amorphous ${\mathrm{Na}}_{2}{\mathrm{Si}}_{2}O_{5}$ solid electrolyte compared to crystalline ${\mathrm{Na}}_{2}{\mathrm{Si}}_{2}O_{5}$, although there could be some differences between inorganic solid and polymer electrolytes. The material ${\mathrm{Na}}_{2}{\mathrm{Si}}_{2}O_{5}$ is important because ${\mathrm{Sr}}_{1-x}{\mathrm{Na}}_{x}{\mathrm{SiO}}_{3-0.5x}$, which has relatively high ionic conductivity of $10^{-2}{\;S\;\mathrm{cm}}^{-1}$ at 500 °C, consists of an amorphous ${\mathrm{Na}}_{2}{\mathrm{Si}}_{2}O_{5}$ phase and a ${\mathrm{SrSiO}}_{3}$ phase [104,105,106]. The ionic conduction is only in the amorphous ${\mathrm{Na}}_{2}{\mathrm{Si}}_{2}O_{5}$ phase because the ${\mathrm{SrSiO}}_{3}$ phase is an electric insulator. Lei et al. [104] performed molecular dynamics simulations to study the mechanism of high ionic conductivity in the amorphous ${\mathrm{Na}}_{2}{\mathrm{Si}}_{2}O_{5}$ phase (Figure 14). The structures of crystalline and amorphous ${\mathrm{Na}}_{2}{\mathrm{Si}}_{2}O_{5}$ used in the molecular dynamics simulations are shown in Figure 14A [104]. The simulated transport of ${\mathrm{Na}}^{+}$ ion in amorphous Na_2_Si_2_O_5_ is shown in Figure 14B, where the ${\mathrm{Na}}^{+}$ ion in motion is shown with a green ball. According to the simulation, the motions of $O^{2-}$ and ${\mathrm{Si}}^{4+}$ are negligible compared to that of the ${\mathrm{Na}}^{+}$ ion. As seen in Figure 14B, the motion of the ${\mathrm{Na}}^{+}$ ion is relatively fast, which supports the high ionic conductivity in the amorphous Na_2_Si_2_O_5_ phase. On the other hand, the lowest energy barrier for the ${\mathrm{Na}}^{+}$ ion conduction in the crystalline ${\mathrm{Na}}_{2}{\mathrm{Si}}_{2}O_{5}$ phase is estimated to be as high as 1.18 eV, which is shown in Figure 14C [104]. At such a high energy barrier, the crystalline ${\mathrm{Na}}_{2}{\mathrm{Si}}_{2}O_{5}$ phase would be an electrical insulator [104]. The reason for the high ionic conductivity in the amorphous phase is the much weaker Na-O Coulombic attraction due to the long-range disorder in the amorphous phase comparted to that in the crystalline phase [104].

**Figure 15:**
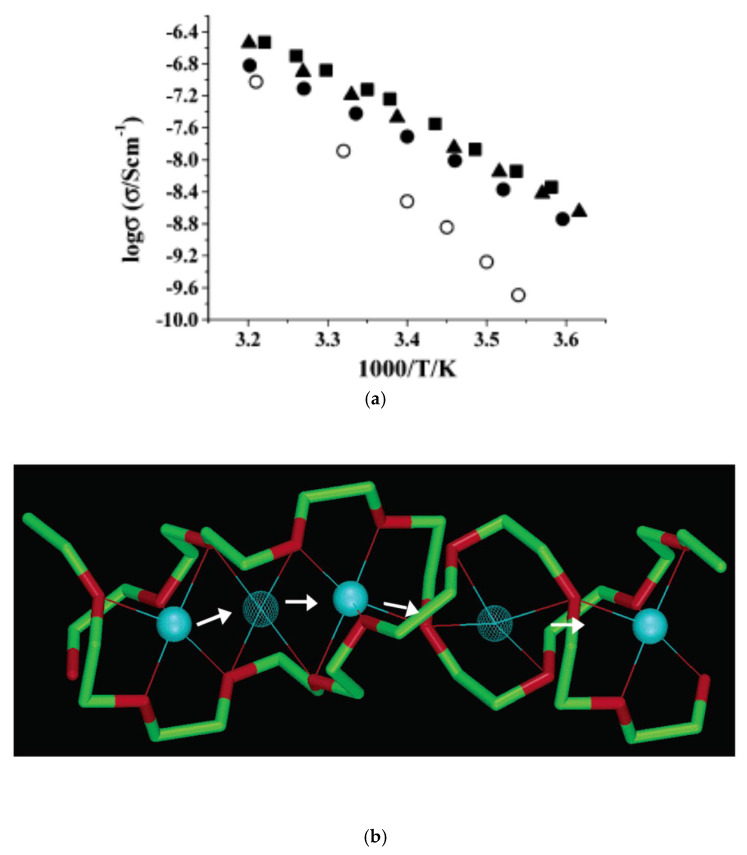
(**a**) Ionic conductivity $\sigma \;\left({S\;\mathrm{cm}}^{-1}\right)$ of crystalline polymer electrolytes ${\mathrm{PEO}}_{6}:{\mathrm{LiPF}}_{6}$ (solid circles), ${\mathrm{PEO}}_{6}:{\mathrm{LiAsF}}_{6}\;$(squares), ${\mathrm{PEO}}_{6}:{\mathrm{LiSbF}}_{6}$ (triangles), and amorphous ${\mathrm{PEO}}_{6}:{\mathrm{LiSbF}}_{6}$ (open circles). (**b**) Schematic diffusion pathway of ${\mathrm{Li}}^{+}$ cations along the polymer tunnel in crystalline ${\mathrm{PEO}}_{6}:{\mathrm{LiPF}}_{6}$. The blue solid spheres show a ${\mathrm{Li}}^{+}$ cation in the crystallographic five-coordinate site where the thin lines show the coordination. The meshed blue spheres show a ${\mathrm{Li}}^{+}$ cation in the intermediate four-coordinate site where green and red show carbon and oxygen, respectively. Reprinted with permission from Ref. [51]. Copyright 2003, American Chemical Society.

As already noted in the Introduction, however, there are some experimental and theoretical reports that ionic conductivity in crystalline polymer electrolytes could be higher than in amorphous polymer electrolytes [50,51,52]. As seen in Figure 15a, experimentally measured ionic conductivity of crystalline polymer electrolytes is considerably higher than that of the amorphous polymer electrolytes [51]. The ionic conductivity was determined by plotting complex impedance (Cole–Cole plot) obtained by ac impedance measurements [51]. The data in Figure 15a for amorphous $P\left({\mathrm{EO}}_{6}\cdot {\mathrm{LiSbF}}_{6}\right)$ are all above the glass transition temperature ($T_{g}=-33\;{}^{\circ}C$), which means that the amorphous material is in the conducting state. From the gradient of the curves for crystalline polymer electrolytes in Figure 15a, the activation energy is derived as about 1.0 eV which is slightly smaller than that (1.18 eV) estimated by ab initio molecular dynamics simulations for crystalline ${\mathrm{Na}}_{2}{\mathrm{Si}}_{2}O_{5}$ solid electrolyte, which would be an electrical insulator, by Lei et al. [104]. In other words, the ionic conductivity shown in Figure 15a is relatively very low both for crystalline and amorphous polymer electrolytes.

The diffusion pathway for the faster ionic conduction along the polymer tunnel in crystalline polymer electrolytes, compared to that in amorphous polymer electrolytes, is schematically shown in Figure 15b [51]. The ${\mathrm{Li}}^{+}$ conduction along polymer tunnel is described by the jump-diffusion model discussed in Section 3.3 because ${\mathrm{Li}}^{+}$ ion jumps from the crystallographic five-coordinate site (solid blue spheres in Figure 15b) to the intermediate four-coordinate site (meshed blue spheres), and so on. Indeed, the temperature dependence of ionic conductivity of crystalline polymer electrolytes in Figure 15a is the Arrhenius type (linear relationship in the Arrhenius plot) [51]. The ${\mathrm{Li}}^{+}$ ion conduction along the polymer tunnels shown in Figure 15b could only occur, however, when there are a sufficient number of vacant ${\mathrm{Li}}^{+}$ sites which are necessary for ${\mathrm{Li}}^{+}$ ions to migrate [51].

**Figure 16:**
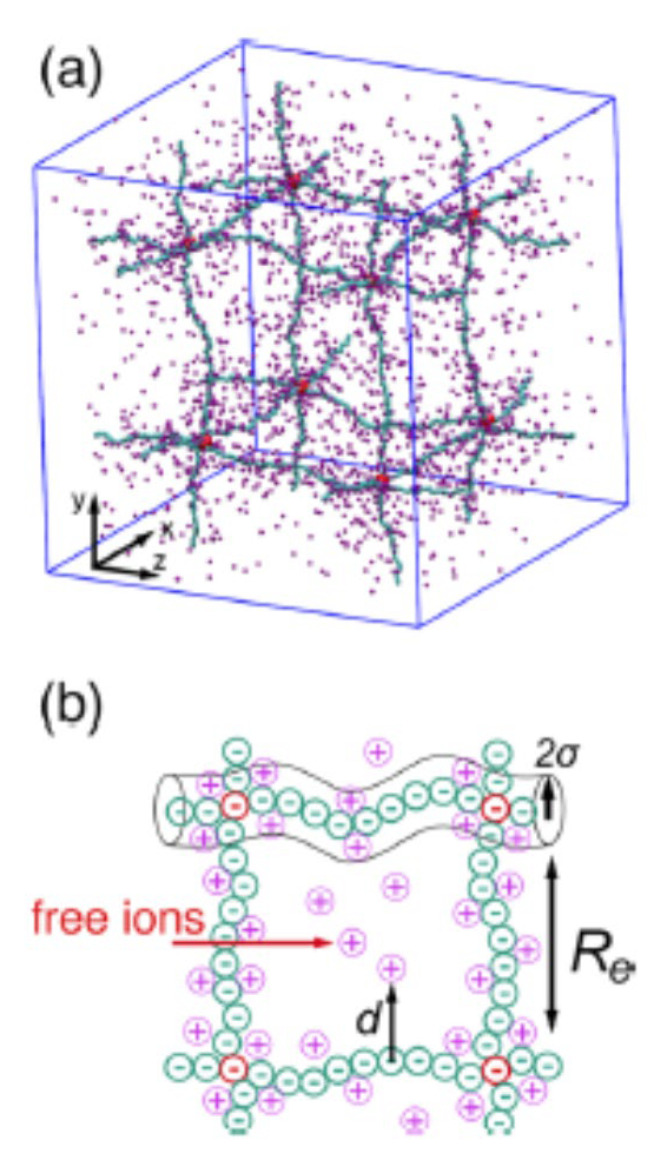
(**a**) Snapshot depicting the unit lattice of an N = 100 isotropic polyelectrolyte network structure in a swollen polyelectrolyte hydrogel by molecular dynamics simulations. Monomers and counterions are denoted by cyan and purple spheres, respectively. Each cross-linking node is attached by six polyelectrolyte chains, each of which has N monomers. (**b**) Free ions apart from the gel backbone can move faster. Reprinted with permission from Ref. [107]. Copyright 2016, American Chemical Society.

Finally, the mechanism for ionic conduction in polymeric gel electrolytes is discussed based on the coarse-grained molecular dynamics simulations by Li et al. [107]. In Figure 16a, the unit cell of a defect-free isotropic cubic polyelectrolyte network is shown, which is used in the simulations [107]. The polymer segments connecting two network nodes are modeled by bead-spring chains composed of N monomers (N = 100 in Figure 16) [107]. In this case, monomers (cyan in the figure) are negatively charged, and the counterions (purple) are positively charged. When a counterion is sufficiently apart from the polymer chains, the mobility, which is proportional to the ionic conductivity if ion–ion interaction is not so strong [108,109], becomes relatively high. Such an ion is called a free ion [107]. Some other ions are close to the chains but they are still mobile. The other ions are condensed on the chains due to the electrostatic attraction, which are immobile. As the applied electric field is increased, the fraction of mobile ions increases, resulting in an increase in the ionic conductivity of the system [107]. Further studies are required on more detailed mechanisms for ionic conduction in polymeric or inorganic gel electrolytes.

## 5. Methods to Increase Ionic Conductivity

The ionic conductivity of polymer electrolytes is relatively low compared to liquid or inorganic solid electrolytes [93]. The basic methods used to increase the ionic conductivity of soft matter electrolytes are to increase the concentration of mobile ions, and the introduction of large anions with delocalized electron structures for easier dissociation of the salts [110]. For systems with ion transport strongly correlated with polymer dynamics, the design of flexible polymer structures for better mobility of ions is useful, which is sometimes related to the decrease in glass transition temperature or decrease in crystallinity of the polymer matrix [111,112]. When the ion radius is relatively small, such as in the case of ${\mathrm{Li}}^{+}$ ions, a higher dielectric constant of the polymer matrix could increase ionic conductivity because it weakens ion–ion interaction which reduces ionic conductivity under this condition [108]. In the present section, two interesting methods to increase the ionic conductivity of polymer electrolytes are briefly discussed. One is cavitation in polymer electrolytes by tensile deformation [113]. The other is a microporous (or macroporous) structure in polymer electrolytes [114]. In addition, a possible method to increase the ionic conductivity of crystalline electrolytes by introducing high-density dislocations is briefly discussed [115,116].

### 5.1. Cavitation in Polymer Electrolytes (Experiments)

**Figure 17:**
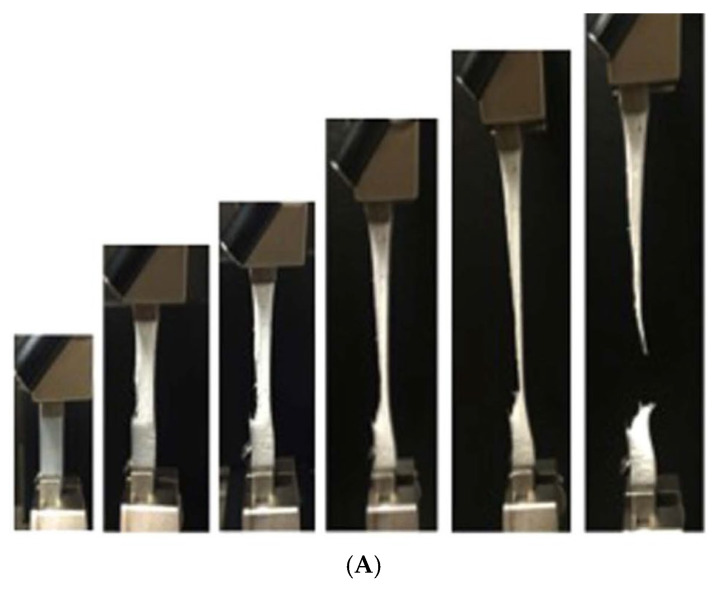

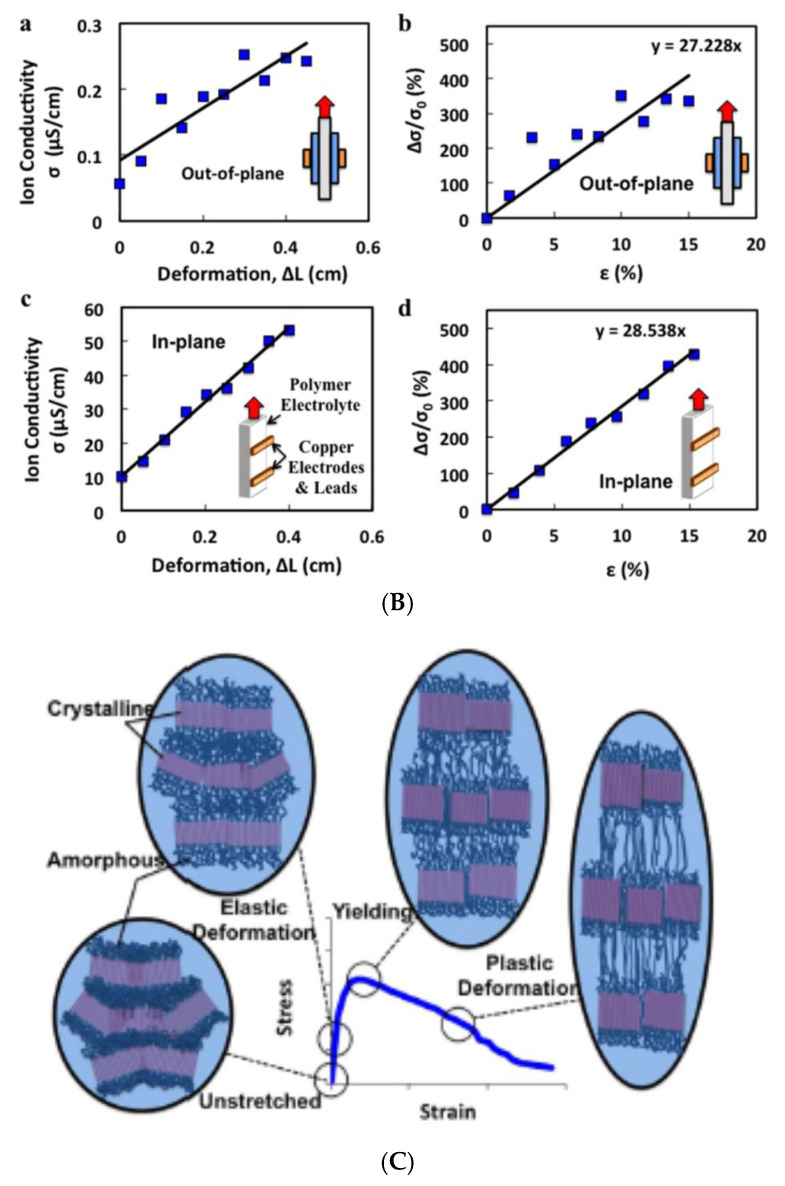
(**A**) Photo images of PEO samples subjected to tensile deformation. (**B**) In-plane and out-of-plane ionic conductivities of PEO electrolyte (soft matter electrolyte) with respect to tensile deformation (in the direction of the red arrow). (**a**) Out-of-plane ionic conductivity vs. tensile deformation of PEO/Li salt film. (**b**) Out-of-plane enhancement in ionic conductivity vs. tensile strain. (**c**) In-plane ionic conductivity vs. tensile deformation. (**d**) In-plane enhancement in ionic conductivity vs. tensile strain. (**C**) Depiction of semi-crystalline polymer microstructure at various stages of tensile deformation. Reprinted with permission from Ref. [113]. Copyright 2016, Kelly et al.

Cavitation in polymer specimens under tensile deformation leads to the formation of many voids in the material [117,118,119,120,121,122]. This phenomenon is somewhat different from the widely known acoustic (ultrasonic) or hydrodynamic cavitation in liquids, where the created “voids”, which are gas and vapor bubbles in this case, violently collapse, resulting in high temperatures and pressures inside the bubbles [123,124,125,126,127,128,129,130,131,132,133]. As polymer specimens are not fluids, created voids in polymer cavitation do not violently collapse in contrast to the cases of acoustic or hydrodynamic cavitation. Nevertheless, created voids play a considerable role in increasing the ionic conductivity of polymer electrolytes, as shown in Figure 17 [113].

In the experiments shown in Figure 17, thin-film semicrystalline polymer electrolyte (PEO):${\mathrm{LiClO}}_{4}$ with a thickness of about 200–300 μm was used [113]. In Figure 17A, photo images of the polymer electrolyte subjected to tensile deformation are shown. Under the tensile strain rate of 3.5 mm/min, rupture of the specimen occurred at about 450% in strain [113]. The yield stress was about 1.2 MPa when the tensile strain was about 5% [113]. In Figure 17B, the measured ionic conductivity at room temperature is shown as a function of the magnitude of tensile deformation or strain [113]. It is clearly seen that ionic conductivity considerably increases as the magnitude of deformation as well as strain is increased [113]. The increasing rates in in-plane and out-of-plane ionic conductivities are nearly the same, although the absolute value of in-plane ionic conductivity is significantly higher than the out-of-plane conductivity, as seen in Figure 17B [113]. In other words, the effect of tensile deformation on ionic conductivity is nearly isotropic [113].

As the tensile deformation proceeds, tiny voids are expected to be created especially in the amorphous regions of the semicrystalline polymer electrolyte (Figure 1 and Figure 17C), which is cavitation. According to the free volume model discussed in Section 3.1, the creation of tiny voids could correspond to the increase in the free volume for ionic conduction (Figure 13), resulting in an increase in ionic conductivity (Equation (15)). According to the configurational entropy model discussed in Section 3.2, the creation of tiny voids could imply that cooperative rearrangements of polymer molecules become easier, resulting in an increase in ionic conductivity. This is possibly due to the decrease in the change of chemical potential of the molecules in the cooperative arrangement (Δμ) in Equation (24).

**Figure 18:**
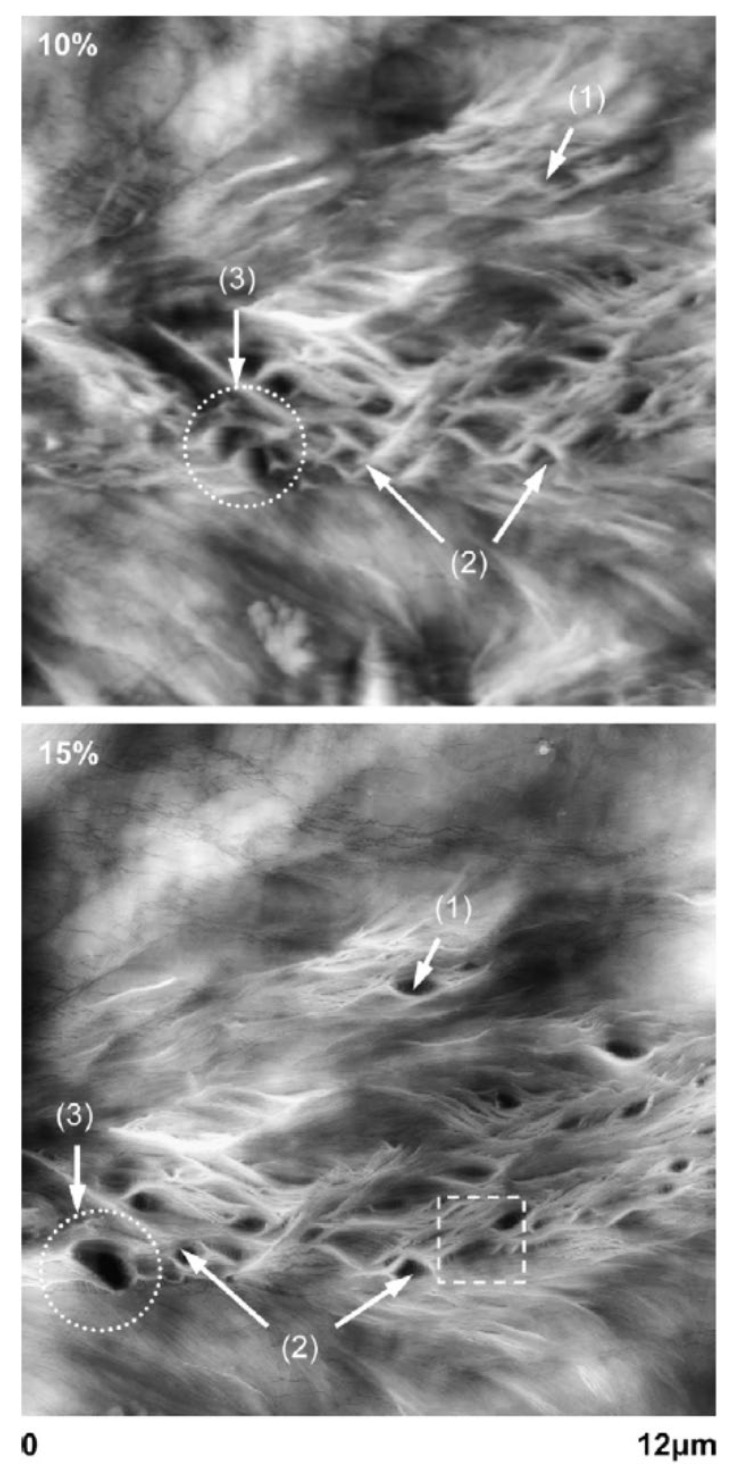
AFM height images of equatorial region of a polybutene spherulite (semi-crystalline polymer) for two strain levels 10 and 15%. The void formation (1), the growth (2), and the coalescence (3) of cavities are indicated in the images. Reprinted with permission from Ref. [118]. Copyright 2007, Elsevier.

Some examples of AFM images for tiny voids created by tensile deformation (cavitation) are shown in Figure 18 for PB films with thicknesses of about 100 μm under tensile strains of 10% (upper figure) and 15% (lower figure) [118]. The used PB films are made of poly(1-butene) with the number- and weight-average molar weight of 28 kDa and 174 kDa, respectively [118]. The tiny voids are shown in Figure 18 with some arrows as follows: (1) the opening, (2) the growth, and (3) the coalescence of voids [118]. The size of the voids in the cavitation is from a submicron to a few microns, as shown in Figure 18 [118]. Further studies are required on the details of cavitation in various polymer materials. Recently, Jeanne-Brou et al. [134] also experimentally reported an increase in the ionic conductivity of solid polymer electrolytes by tensile deformation.

### 5.2. Microporous (or Macroporous) Composite Polymer Electrolytes (Experiments)

Here, “microporous” means that the typical pore diameter is less than about 30 μm, which is also called “macroporous”. As seen in the previous subsection, the presence of tiny voids from a submicron to a few microns in diameter in semicrystalline polymer electrolytes could considerably increase ionic conductivity. The tiny voids are created by tensile deformation in the previous subsection, which is cavitation in polymer materials. In the present subsection, pores (voids) are introduced by adding a filler (mesoporous silica with pore sizes up to about 30 nm) to polymer electrolytes [114].

**Figure 19:**
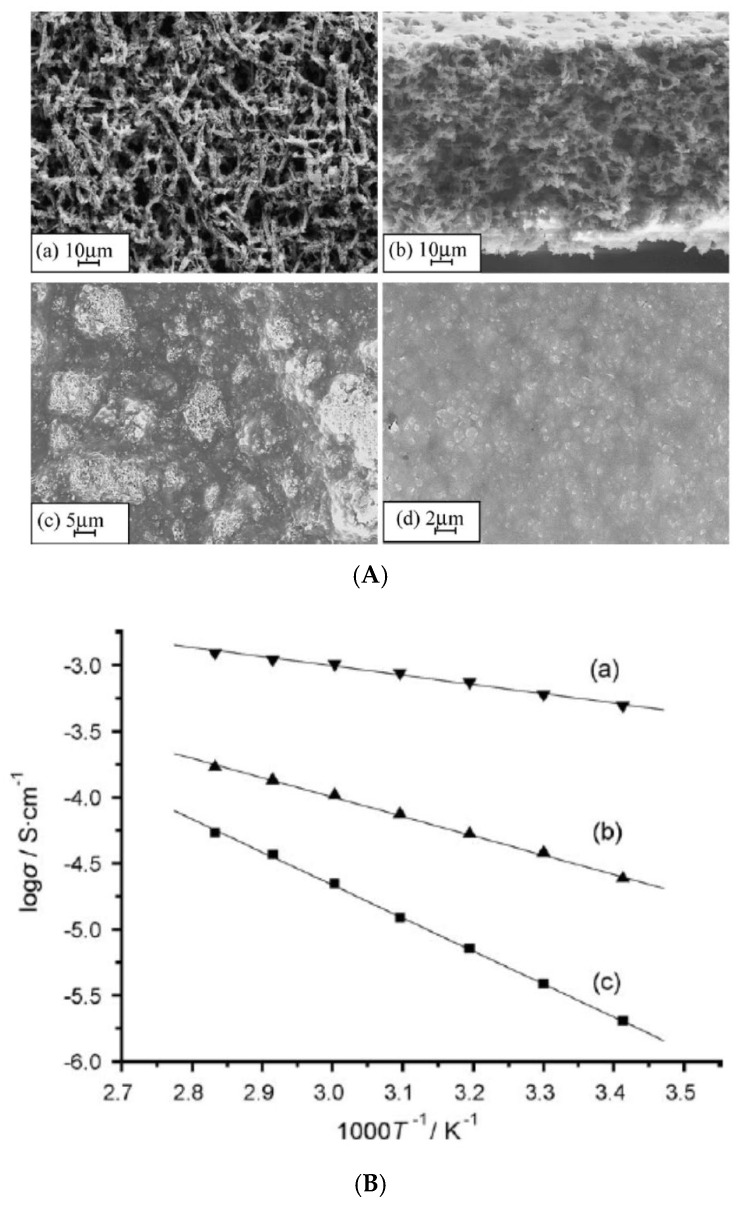
(**A**) SEM images of composite polymer membranes with different molecule sieves: (**a**) 0.15 g SBA-15 (silica with micro- and narrow mesopores), with rich pores; (**b**) its cross-section; (**c**) 0.15 g MCM-41 (another form of silica), without any pores; (**d**) 0.15 g NaY, without any pores. (**B**) Arrhenius plots of ionic conductivity for the composite polymer electrolyte (PVdF-HFP/${\mathrm{LiPF}}_{6}$) films of (**a**) 0.15 g SBA-15; (**b**) 0.15 g MCM-41; (**c**) 0.15 g NaY. Reprinted with permission from Ref. [114]. Copyright 2006, Elsevier.

In the experiments shown in Figure 19 [114], microporous composite electrolytes are synthesized by adding SBA-15 (150(d) nm × 400(h) nm particle size) [135], which is a mesoporous silica with pore sizes of up to 30 nm, to the polymer electrolyte PVdF-HFP (poly(vinylidene fluoride-co-hexafluoropropylene) which is a copolymer). In the synthesis, powders of the filler and the copolymer are dissolved in the organic solvent DMF (N,N-dimethylformamide, ${\left({\mathrm{CH}}_{3}\right)}_{2}\mathrm{NCHO}$) [114]. The composite film is formed by drying the mixture on a smooth cleaned glass plate [114]. During the drying process, the solvent DMF is concentrated on the surfaces of the filler SBA-15 where voids (pores) are formed, while the other polymer-rich parts become solid phase after drying [114,136]. In this way, a microporous structure is formed. Finally, an electrolyte solution of ${\mathrm{LiPF}}_{6}$ is added to the film, and the microporous composite polymer electrolyte is formed [114]. Other composite polymer electrolytes are also made using NaY (500 nm particle size) or MCM-41 [137] (100(d) nm × 300(h) nm particle size, which is another mesoporous silica with smaller pore sizes than those of SBA-15) as a filler [114].

The composite polymer electrolyte with SBA-15 has a microporous structure with a pore size of less than 10 μm, as shown in Figure 19A(a,b) [114]. On the other hand, the composite polymer electrolytes with MCM-41 and NaY scarcely have pores, as shown in Figure 19A(c,d), respectively [114]. The ionic conductivity of the microporous composite polymer electrolyte with SBA-15 is as high as 0.5 mS ${\mathrm{cm}}^{-1}$ at room temperature, which is about 2–3 orders of magnitude larger than that of the film without any fillers [114]. Furthermore, the microporous composite polymer electrolyte with SBA-15 has a considerably higher ionic conductivity than that of MCM-41 or NaY without pores, as shown in Figure 19B [114]. It may be possible that micropores work as free volume, as discussed in Section 3.1, or that they make the cooperative rearrangements of polymer molecules for ionic conduction much easier, as already pointed out in the previous subsection. However, the temperature dependence of ionic conductivity of the composite polymer electrolytes seems to be the Arrhenius type. as seen in Figure 19B, which suggests the jump-diffusion model holds rather than the free volume or configurational entropy model. Further studies are required on the mechanism of the increase in ionic conductivity in the microporous composite polymer electrolyte. There are also some other experimental reports on the increase in ionic conductivity by adding a filler to polymer electrolytes [138,139,140].

### 5.3. All-Dislocation-Ceramics in Solid Electrolytes (Theory)

In the present subsection, a possible method to increase the ionic conductivity of crystalline ceramic electrolytes proposed by the authors is discussed [115]. At the end of the subsection, the possibility of application to semicrystalline polymer electrolytes is briefly discussed. The method involves introducing high-density dislocations into crystalline electrolytes because ionic conductivity along a dislocation is several orders of magnitude higher than that in the bulk [115,141,142]. The reason for the higher ionic conductivity along a dislocation is the considerably lower formation energy of a vacancy in the dislocation pipe, which is wider than the dislocation core of about 1 nm in diameter [143,144,145,146]. The width of the dislocation pipe is related to the width of the space charge region around the positively charged dislocation [144]. The typical diameter of a dislocation pipe is about 3 nm [143,145,146]. It should be noted, however, that ionic conductivity *across* a dislocation is even lower than in the bulk [147].

As the ionic conductivity along a dislocation pipe is several orders of magnitude higher than in the bulk, dendrite formation along a dislocation is inevitable. If the electrode surface is completely covered with the cross sections of many dislocation pipes, dendrite formation would be avoided because ionic current density becomes spatially uniform [115]. In other words, if the solid electrolyte is filled with parallel straight dislocation pipes, dendrite formation could be avoided. Such ceramic electrolytes are called all-dislocation-ceramics [116,148,149].

**Figure 20:**
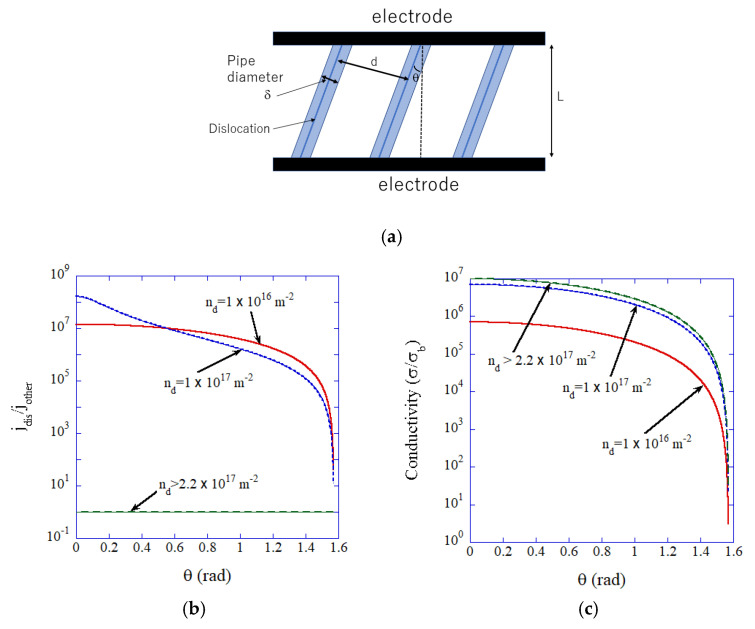
(**a**) Model of single-crystal solid electrolyte with parallel dislocations. (**b**) Calculated spatial variation of ionic current density ($\frac{j_{dis}}{j_{other}},\;\mathrm{where}\;j_{dis}\;\mathrm{and}\;j_{other}\;\mathrm{is}$ ionic current density along dislocations and in other regions, respectively), as a function of angle (θ) for various dislocation densities ($n_{d}$). (**c**) Calculated mean ionic conductivity relative to the bulk ionic conductivity ($\sigma /\sigma_{b}$). Reprinted with permission from Ref. [115]. Copyright 2023, IOP Publishing Ltd.

In Figure 20, the results of numerical calculations of spatial variation in ionic current density, as well as mean ionic conductivity, are shown [115]. In the calculations, parallel straight dislocations are considered as in Figure 20a [115]. When the diameter of a dislocation pipe is 3 nm, the condition for all-dislocation-ceramics is expressed by a dislocation density higher than about $2.2\times 10^{17}\;m^{-2}$ [115]. Under this condition, the ionic current density becomes nearly spatially uniform (Figure 20b), and the mean ionic conductivity becomes several orders of magnitude higher than that of the bulk (Figure 20c) [115]. The equations used in the numerical calculations are described in Reference [115].

**Figure 21:**
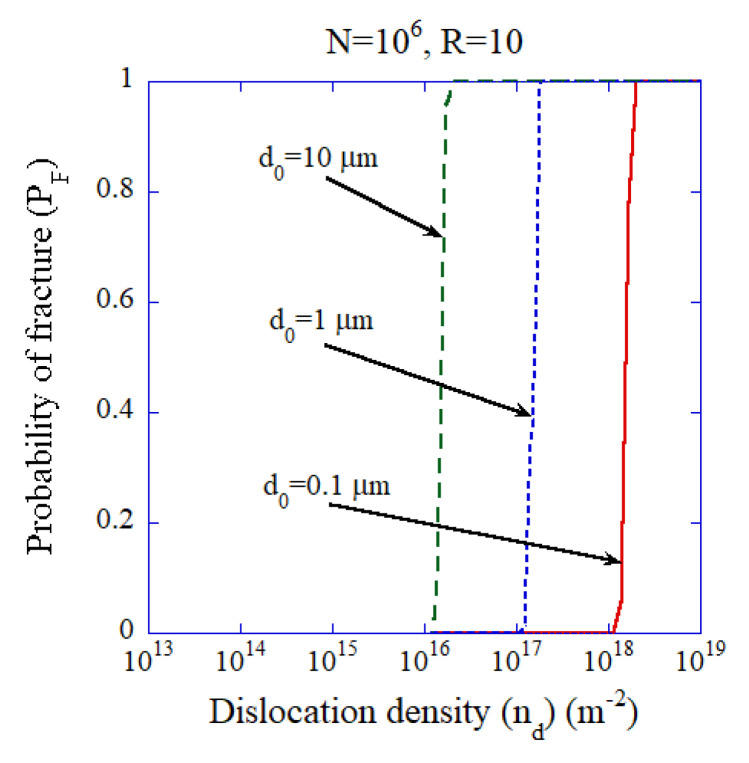
The results of numerical calculations for probability of fracture ($P_{F}$) as a function of dislocation density when the number of microcracks is $N=10^{6}$ for various values of the characteristic diameter of pre-existing microcracks ($d_{0}$). R is the ratio of the compressive strength to the tensile strength (R=10 is assumed). Reprinted with permission from Ref. [150]. Copyright 2023, IOP Publishing Ltd.

As ceramic materials are brittle, there is a possibility that crystalline ceramic electrolytes will be fractured during the introduction of high-density dislocations. In order to study this possibility, numerical calculations of the probability of the fracture of a ceramic specimen as a function of dislocation density are performed (Figure 21) [150]. The compressive strength of a specimen is determined by the diameter of the largest pre-existing microcrack in the specimen according to the Griffith criterion for fracture [150,151]. The diameter of the largest pre-existing microcrack is determined statistically using a probability model under a given size distribution of pre-existing microcracks in a ceramic specimen [150]. The dislocation density introduced into the specimen is related to the applied stress through the Bailey–Hirsch type relationship [150,152]. Using the compressive strength calculated with a probability model, the probability of fracture is calculated as a function of introduced dislocation density as shown in Figure 21 [150]. When the characteristic diameter of the pre-existing microcrack which determines the size distribution of pre-existing microcracks is sufficiently small (smaller than about 1 μm), a dislocation density as high as about $10^{17}{\;m}^{-2}$ is achievable without fracture of the specimen (Figure 21) [150].

**Figure 22:**
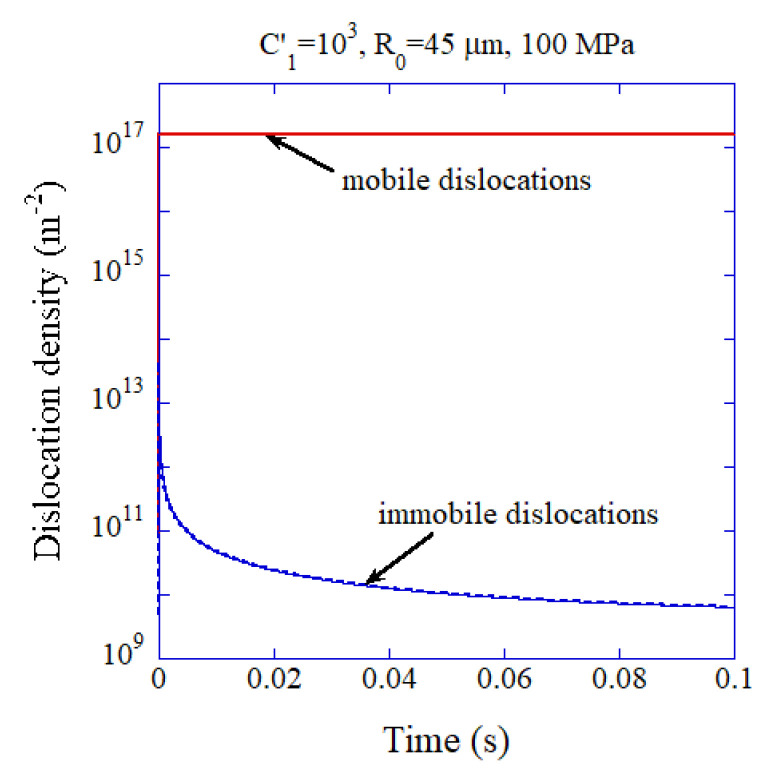
The results of numerical simulations on the mobile- and immobile-dislocation densities as a function of time during dry pressing of LATP (solid electrolyte) particles with the initial radius of $R_{0}=45\;\mu m$ under the applied pressure of 100 MPa. *Ć*_1_ is the parameter related to the multiplication of mobile dislocations (*Ć*_1_$=10^{3}$ is assumed). Reprinted with permission from Ref. [148]. Copyright 2024, Yasui et al.

In many experiments of dislocation engineering of ceramic materials to improve their functional, electrical, and mechanical properties, dislocations are introduced into ceramic materials by applying compressive stress at room temperature or elevated temperatures [153,154,155]. The authors [148] investigated the possibility of the introduction of high-density dislocations into solid electrolytes during dry pressing by numerical simulations of the evolution of mobile and immobile dislocations (Figure 22). Dry pressing is a consolidation process of ceramic particles by applying high compressive stress [156]. The temperature for dry pressing is relatively low, such as 300 °C or less as in the case of cold sintering [156,157,158,159]. The difference between dry pressing and cold sintering is the use of liquid (water) in cold sintering [156,157,158]. Cold sintering and dry pressing have been widely studied because they are beneficial to save energy and reduce CO_2_ emissions due to the relatively low temperatures for sintering [156,157,158,159]. The results of the numerical simulation shown in Figure 22 suggest that the high-density dislocations above $10^{17}{\;m}^{-2}$ required for all-dislocation-ceramics are achievable under some dry pressing conditions [148]. There have been already some experimental reports on the generation of dislocations in cold sintering [160,161,162]. Further studies are required to produce all-dislocation-ceramics by dry pressing or cold sintering. Some experimental results suggest that all-dislocation-ceramics could have higher fracture toughness, which is beneficial to prevent dendrite formation as well as to avoid rupture [116,163,164,165]. Fracture toughness is the resistance to crack propagation, which is an important property for engineering materials to prevent rupture [116,151].

With regard to the application to semicrystalline polymer electrolytes, dislocations in the crystalline phase are actually present and could increase ionic conductivity, as in the case of ceramic electrolytes according to some theoretical and experimental reports [166,167,168,169]. Further studies are required on whether high-density dislocations could possibly increase ionic conductivity without dendrite formation in semicrystalline polymer electrolytes.

## 6. Merits and Demerits of Soft Matter Electrolytes

As noted in the Introduction, soft matter electrolytes are defined in the present review as polymer electrolytes and polymeric or inorganic gel electrolytes (Figure 1). Polymer electrolytes are mixtures of a polymer and Li salt(s) or polymerized ionic liquids. A more strict definition of soft matter electrolytes is provided, however, by Young’s modulus of electrolytes: from $10^{5}$ Pa to $10^{9}$ Pa (Table 1) [6,38,39,40,41,170]. A lower value of Young’s modulus corresponds to a softer material [35]. Young’s modulus for solid electrolytes is from about $10^{10}$ Pa to $10^{11}$ Pa [42], as already noted in the Introduction. The bulk modulus of liquid electrolytes is about $10^{9}$ Pa [43,44]. Liquid electrolytes, which are organic solutions of Li salts, have been widely used in conventional Li-ion batteries [1,2,3]. The ionic conductivity of typical liquid electrolytes is in the order of $10^{-2}$ S ${\mathrm{cm}}^{-1}$ at room temperature, which is typically higher than those of solid or soft matter electrolytes [93]. It should be noted that for some solid or gel electrolytes, ionic conductivity is comparable to that of liquid electrolytes [25,93,171]. For liquid electrolytes, however, there is a safety problem in that they can possibly leak and are burnable by vaporization, especially when batteries are heated or short-circuited by dendrite formation (Table 1) [2,4]. Solid electrolytes could solve the safety problem because they are not burnable without any leakage [4,5]. However, solid electrolytes have another problem, as the contact between the electrodes and solid electrolytes is rather poor (Table 1) [6]. On the other hand, soft matter electrolytes could have much better contact with electrodes [6,59]. In addition, leakage may not occur, although some soft matter electrolytes are still burnable [6]. The problem with soft matter electrolytes is their relatively low ionic conductivity at room temperature, except for some gel electrolytes which could have conversely insufficient mechanical properties as separators in Li-ion batteries [59,93]. There are some reports that the mechanical properties of polymeric gel electrolytes could be improved by adding fumed silica particles [172]. Another problem of soft matter electrolytes is the relatively high possibility of degradation and aging [95,173,174]. Degradation (aging) of polymer materials has been widely reported, which could decrease the ionic conductivity [95,173,175,176]. With regard to liquid or solid electrolytes, degradation may occur mostly at the interface between electrodes and electrolytes where the interface layer is formed, which increases the electrical resistance [177,178]. Another merit of soft matter electrolytes is their relatively high mechanical flexibility, which is the ability of a material to deform elastically and return to its original shape when the applied stress is removed. Accordingly, soft matter electrolytes could be used in flexible batteries [179].

Finally, the ${\mathrm{Li}}^{+}$ transference number in Table 1 is discussed. Here, we consider a salt MX which is dissociated into $M^{+}$*,* $X^{-}$, ${\left[M_{2}X\right]}^{+}$, and ${\left[{\mathrm{MX}}_{2}\right]}^{-}$ [11]. The transport number ($t_{i}$), which is in general different from the transference number ($T_{i}$), of any of the charged species, is the proportion of electrical current carried by that species under an applied electric field [11]. Thus, the sum of the transport numbers for all charged species present is unity. On the other hand, the transference number is the proportion of electrical current carried by a salt constituent, as follows [11]:

(26) $$T_{X^{-}}=t_{X^{-}}+2t_{M-}-t_{M_{2}X^{+}}$$

(27) $$T_{M^{+}}=t_{M^{+}}+2t_{M_{2}X^{+}}-t_{MX_{2}^{-}}$$

The sum of $T_{X^{-}}$ and $T_{M^{+}}$ is unity [11]. The transference number ($T_{i}$) and the transport number ($t_{i}$) are equal when the electrolyte is dissociated into two ionic species $M^{+}$ and $X^{-}$ [11]. For the performance of Li-ion batteries, a higher ${\mathrm{Li}}^{+}$ transference number as well as transport number is preferable. The ${\mathrm{Li}}^{+}$ transference number is relatively high (nearly unity) for solid electrolytes, as most of the electrical current is carried by ${\mathrm{Li}}^{+}$ ions. On the other hand, the ${\mathrm{Li}}^{+}$ transference number in liquid or soft matter electrolytes could be considerably lower, as anions also carry some electrical current [1,180,181,182,183,184,185].

## 7. Conclusions

In the present review, soft matter electrolytes are defined as polymer electrolytes and polymeric or inorganic gel electrolytes. They are defined more strictly by Young’s modulus from about $10^{5}$ Pa to $10^{9}$ Pa, where a lower value corresponds to a softer material. Many soft matter electrolytes exhibit VFT (Vogel–Fulcher–Tammann)-type temperature dependence of ionic conductivity (Equation (6) or (7)). VFT-type behavior is explained by the free volume model or the configurational entropy model, which is discussed in detail. Mostly, ionic conduction in polymer electrolytes is through the amorphous phase of the polymer. There are, however, some experimental and theoretical reports that the crystalline phase of polymer is a better ionic conductor than the amorphous phase. Some interesting methods to increase the ionic conductivity of polymer electrolytes are discussed, such as cavitation under tensile deformation and the microporous structure of polymer electrolytes. The merits and demerits of soft matter electrolytes are discussed, comparing them with liquid and solid electrolytes. The merits are safer properties compared to liquid electrolytes, as well as more mechanical flexibility. The demerits are relatively low ionic conductivity at room temperature when mechanical properties are relatively good, and more possible degradation by aging compared to liquid or solid electrolytes.

## Figures and Tables

**Table 1:** Comparison between soft matter, liquid, and solid electrolytes for ${\mathrm{Li}}^{+}$ ion conduction.

	Soft Matter Electrolytes	Liquid Electrolytes	Solid Electrolytes
Materials	Li Salt in Polymer/Gel	Li Salt in Organic Solvent	Ceramics
Young’s modulus (Pa) (Softness)	$10^{5}\sim 10^{9}$	$10^{9}$ (Bulk modulus)	$10^{10}\sim 10^{11}$
Ionic Conductivity	Low~Medium	High	Medium
Li+ Transference Num.	Low~Medium	Low~Medium	High
Mechanical Flexibility	High	Low	Medium
Contact at Electrodes	Good	Excellent	Poor
Degradation (Aging)	Highly Possible	Possible (Interfaces)	Possible (Interfaces)
Leakage	Less Possible	Highly Possible	None
Burnability	Low~Medium	High	None

## Data Availability

No new data were created or analyzed in this study.
